# Supplementary material for: Missing science: A scoping study of COVID-19 epidemiological data in the United States
Source: PLoS One. 2022 Oct 12;17(10):e0248793. doi: 10.1371/journal.pone.0248793 (PMC9555641; doi:10.1371/journal.pone.0248793)
Supplement: S1 Protocol — (PDF) [file pone.0248793.s001.pdf]

S1 Protocol

Epidemiological data for COVID-19 pandemic management in the United States: a protocol for a scoping review

Registration: Unregistered

AUTHORS

Rajiv Bhatia
Stanford University
drajiv@stanford.edu

Isabella Sledge

Contributions: Equal among authors

Contact: Rajiv Bhatia

Support: No sponsors or external funding or financial support

INTRODUCTION

Rationale: Systematic assessment of the completeness and timeliness of epidemiological data can inform current and future disease management research responses.

Objectives: Our objective is to assess the scope and completeness epidemiological research conducted in the United States during the first 18 months of the COVID-19 pandemic. More specifically, we aim to examine the scope of research conducted by or with governmental public health entities in U.S. domestic setting and populations informing knowledge on infectious transmission, severity, or disease burden in the pre-vaccine era.

METHODS

Study and Report Eligibility Criteria

Population:	Any population or sub-population.
Setting:	Any domestic community or institutional setting in the United States.
Study Timeframe:	Any timeframe

Study Outcomes:	The review will include descriptive studies enumerating incident COVID-19 infections and describing person, place, or time characteristics of these infections, including case series, cluster or outbreak investigations, and incidence report; analytic studies producing quantitative estimates of transmission parameters (i.e., growth rate or reproductive number, specific attack rates, generation time or serial interval), severity measures (i.e., symptomatic fractions, infection hospitalization or fatality ratios, case hospitalization or fatality ratios, or hospital fatality ratios), disease burden measures (incidence of infections, cases, hospitalizations, or fatal outcomes), and measures of excess deaths; and analytic reports producing effect measures for determinants of transmission, disease severity, or disease burden measures.<br><br>The review will exclude studies whose outcomes exclusively concern population behaviors (e.g., mobility), effects of clinical and pharmacological management, effects of vaccines, or indirect effects of pandemic responses.
Study Design:	This review will include observational epidemiologic studies of human populations using any study design.<br><br>This review will exclude laboratory, phylogenetic, and environmental studies, synthetic modeling exercises, and studies describing infection control plans or protocols.
Report Publication Status:	Any study published and available via electronic media before November 30, 2021.
Report Language:	English
Report Authorship:	At least one report author with an institutional affiliation with a U.S governmental public health entity.

Information sources:

The review will primarily utilize PubMed using the search terms below to identify report potentially meeting our eligibility criteria. We will additionally screen all reports published in the US CDC publication Morbidity and Mortality Weekly Report during the same time.

Search strategy

The following PubMed search terms will be applied to identify reports for screening.

PubMed Search Terms

(<br>("COVID-19"[Mesh] OR “SARS-CoV-2”[Mesh] OR “COVID-19”)<br>AND<br>("Coronavirus Infections/diagnosis"[Mesh] OR "Coronavirus Infections/epidemiology"[Mesh] OR "Coronavirus Infections/ethnology"[Mesh] OR "Coronavirus Infections/mortality"[Mesh] OR "Coronavirus Infections/prevention and control"[Mesh] OR "Coronavirus Infections/statistics and numerical data"[Mesh] OR "Coronavirus Infections/transmission"[Mesh] OR "COVID-19/epidemiology"[Mesh] OR "COVID-19/mortality"[Mesh] OR "COVID-19/prevention and control"[Mesh] OR "COVID-19/statistics and numerical data"[Mesh] OR "COVID-19/transmission"[Mesh] OR "United States/epidemiology"[Mesh] OR "United States/statistics and numerical data"[Mesh] OR “Contact Tracing”[Mesh] OR "prevention and control" [Subheading] OR "transmission" [Subheading] OR "disease transmission, infectious"[Mesh])<br>AND<br>(“United States” [Mesh])<br>AND<br>(CDC[Affiliation] OR “Centers for Disease Control and Prevention” [Affiliation] OR CDC[Author - Corporate] OR “2019-nCoV CDC Response Team”[Author - Corporate] OR “CDC COVID-19 Response Team” OR “2019-nCoV CDC Response Team” OR "Public Health Department"[Affiliation] OR " Health Department"[Affiliation] "Department of Public Health"[Affiliation] OR " Department of Health"[Affiliation] OR “Public Health”[Affiliation])<br>)
OR
(<br>("COVID-19"[Mesh] OR “SARS-CoV-2”[Mesh] OR “COVID-19”)<br>AND<br>("Coronavirus Infections/diagnosis"[Mesh] OR "Coronavirus Infections/epidemiology"[Mesh] OR "Coronavirus Infections/ethnology"[Mesh] OR "Coronavirus Infections/mortality"[Mesh] OR "Coronavirus Infections/prevention and control"[Mesh] OR "Coronavirus Infections/statistics and numerical data"[Mesh] OR "Coronavirus Infections/transmission"[Mesh] OR "COVID-19/epidemiology"[Mesh] OR "COVID-19/mortality"[Mesh] OR "COVID-19/prevention and control"[Mesh] OR "COVID-19/statistics and numerical data"[Mesh] OR "COVID-19/transmission"[Mesh] OR "United States/epidemiology"[Mesh] OR "United States/statistics and numerical data"[Mesh] OR “Contact Tracing”[Mesh] OR "prevention and control" [Subheading] OR "transmission" [Subheading] OR "disease transmission, infectious"[Mesh])<br>AND<br>(“United States” [Mesh])<br>AND<br>(USA[Affiliation])<br>)
OR
(<br>("COVID-19"[Mesh] OR “SARS-CoV-2”[Mesh] OR “COVID-19”)<br>AND

("MMWR. Morbidity and mortality weekly report"[Journal])<br>)
OR
(<br>("COVID-19"[Mesh] OR “SARS-CoV-2”[Mesh] OR “COVID-19”)<br>AND<br>("Emerging infectious diseases"[Journal])<br>)

Data management

The following mechanism that will be used to manage records and data throughout the review

Selection process

Search results and outputs from primary PubMed search will be documented in a text file along with the data of the search. Output of the primary PubMed search including publication title, date, author affiliations, and abstract will be reviewed independently by two reviewers to identify potentially eligible reports. The PMID for all potentially eligible Two reviewers will next and independently read the full text of all potentially eligible studies to confirm eligibility. Reviewer conclusions regarding eligibility will be compared and differences reconciled through verbal discussion. Reasons for excluding studies found to be potentially eligible in the primary search will be documented.

Data collection process

Two reviewers will independently review each study to identify and categorize variables of interest. Variables abstracted from each included report will be recorded a Microsoft Excel electronic spreadsheet. Each report will have one primary reviewer. A second reviewer will confirm the abstracted data recorded by the first reviewer. Conflicting conclusions will be reconciled among the study team.

Data items

We will seek and abstract the following variables from each included study report

Variable	Definition and application
Report Title	
PMID	Enter the PubMed publication identification number
Publication Date	Enter the date of first publication either electronically or in print
First Author	Enter the last name of first author

Author Affiliations	Identify the study as having one or more of the following: US CDC or other federal public health affiliation; State public health affiliation; local public health affiliation; hospital or healthcare affiliation; academic affiliation; other affiliation
Publication Title	Enter the abbreviated journal title
Report Category	Categorized the report as descriptive or analytic
Study Design	Categorize the report as having one of the following study designs: Case control; case series or cluster; cross-sectional; ecologic; incidence; prospective; retrospective
Study Data Source	Categorize the report as having one of the following primary data sources: Active surveillance program; administrative data; field collected data; medical records; passive surveillance; seroprevalence survey; questionnaire survey; passive surveillance program, vital statistics program
Study Setting	
Study Subpopulation	
Data date	
Analytic Outcomes	Identify whether a report provides any of the following analytic outcomes: reproductive number or growth rate; secondary attack rate; incubation period; serial interval or generation time; symptomatic fraction, infection, or case hospitalization ratio; infection, case, or hospital fatality ratio, incidence of infection, seroprevalence, case status, ED visit, hospitalization, or death; excess death; any predictors of infection incidence; any predictor of disease severity.<br><br>Predictors of infection and severe disease will be subcategorized as age or sex; race, ethnicity or socio-economic status; clinical (co-morbid conditions, smoking status); behavioral (use of masks, social distancing, and related regulatory interventions); occupational (job description, industry, work setting, and related regulatory policies); housing or residence characteristics; environmental characteristics; evidence of prior COVID-19 infection; COVID-19 subvariant; or geo-spatial characteristic (census tract characteristic).

Outcomes and prioritization

This is beyond the scope of this scoping study; the review will identify whether an included study report includes a target outcome but will not seek or extract data on that outcome

Risk of bias in individual studies

This is beyond the scope of this scoping study; the review will not identify or assess possible sources of bias of included reports

Data Synthesis

The review will include simple descriptive statistics (frequencies) for categorical variable based on abstracted study data and a narrative synthesis of the results.
