# Supplementary material for: Missing science: A scoping study of COVID-19 epidemiological data in the United States
Source: PLoS One. 2022 Oct 12;17(10):e0248793. doi: 10.1371/journal.pone.0248793 (PMC9555641; doi:10.1371/journal.pone.0248793)
Supplement: S2 Table — (PDF) [file pone.0248793.s003.pdf]

S3 Table: Abstracted Data From Reports Meeting Scoping Review Inclusion Criteria

PMID	Title	Pubdate	First Author	Public Health Affiliation	Publication	Category	Design	Data	Setting	Subpopulation	LastDataDate
32004427	First Case of 2019 Novel Coronavirus in the United States	1/31/2020	Holshue	C	NEJM	Descriptive	Case series or cluster	Medical records	Community	General population	1/30/2020
32027631	Initial Public Health Response and Interim Clinical Guidance for the 2019 Novel Coronavirus Outbreak — United States, December 31, 2019–February 4, 2020	2/7/2020	Patel	A	MMWR	Descriptive	Case series or cluster	Passive Surveillance	Community	General population	2/4/2020
32053579	Persons Evaluated for 2019 Novel Coronavirus — United States, January 2020	2/14/2020	Bajema	A	MMWR	Descriptive	Case series or cluster	Passive Surveillance	Community	General population	1/31/2020
32106216	Update: Public Health Response to the Coronavirus Disease 2019 Outbreak — United States, February 24, 2020	2/28/2020	Jernigan	A	MMWR	Descriptive	Case series or cluster	Passive Surveillance	Community	General population	2/23/2020
32134909	Active Monitoring of Persons Exposed to Patients with Confirmed COVID-19 — United States, January–February 2020	3/6/2020	Burke	B	MMWR	Analytic	Prospective	Field Data	Community	General population	2/26/2020
32178768	First known person-to-person transmission of severe acute respiratory syndrome coronavirus 2 (SARS-CoV-2) in the USA	3/13/2020	Ghinai	B	Lancet	Descriptive	Case series or cluster	Field Data	Community	General population	1/23/2020
32214079	Severe Outcomes Among Patients with Coronavirus Disease 2019 (COVID-19) — United States, February 12–March 16, 2020	3/27/2020	CDCC19RT	A	MMWR	Descriptive	Case series or cluster	Passive Surveillance	Community	General population	3/16/2020
32214083	COVID-19 in a Long-Term Care Facility — King County, Washington, February 27–March 9, 2020	3/27/2020	McMichael	B	MMWR	Descriptive	Case series or cluster	Field Data	Skilled nursing facility	LCTF residents and staff	3/9/2020
32220208	Epidemiology of Covid-19 in a Long-Term Care Facility in King County, Washington	3/27/2020	McMichael	B	NEJM	Descriptive	Case series or cluster	Field Data	Skilled nursing facility	LCTF residents and staff	3/18/2020
32232421	Community Prevalence of SARS-CoV-2 Among Patients With Influenzalike Illnesses Presenting to a Los Angeles Medical Center in March 2020	5/19/2020	Spellberg	C	JAMA	Descriptive	Case series or cluster	Field Data	Community	General population	3/18/2020
32240123	Preliminary Estimates of the Prevalence of Selected Underlying Health Conditions Among Patients with Coronavirus Disease 2019 — United States, February 12–March 28, 2020	4/3/2020	CDCC19RT	B	MMWR	Descriptive	Case series or cluster	Passive Surveillance	Community	General population	3/28/2020
32240128	Asymptomatic and Presymptomatic SARS-CoV-2 Infections in Residents of a Long-Term Care Skilled Nursing Facility — King County, Washington, March 2020	4/3/2020	Kimball	B	MMWR	Descriptive	Case series or cluster	Field Data	Skilled nursing facility	LCTF residents and staff	3/13/2020
32240285	First Mildly Ill, Nonhospitalized Case of Coronavirus Disease 2019 (COVID-19) Without Viral Transmission in the United States-Maricopa County, Arizona, 2020	7/28/2020	Scott	B	Clin Infect Dis	Descriptive	Case series or cluster	Field Data	Community	General population	1/22/2020
32271724	Rapid Sentinel Surveillance for COVID-19 — Santa Clara County, California, March 2020	4/3/2020	Zwald	B	MMWR	Descriptive	Case series or cluster	Active surveillance	Community	General population	3/14/2020
32271726	Detection of SARS-CoV-2 Among Residents and Staff Members of an Independent and Assisted Living Community for Older Adults — Seattle, Washington, 2020	4/3/2020	Roxby	B	MMWR	Descriptive	Case series or cluster	Field Data	Skilled nursing facility	LCTF residents and staff	3/11/2020
32271728	Coronavirus Disease 2019 in Children — United States, February 12–April 2, 2020	4/3/2020	CDCC19RT	A	MMWR	Descriptive	Case series or cluster	Passive Surveillance	Community	Children and Adolescents	4/2/2020
32298245	Timing of Community Mitigation and Changes in Reported COVID-19 and Community Mobility — Four U.S. Metropolitan Areas, February 26–April 1, 2020	4/17/2020	Lasry	B	MMWR	Descriptive	Incidence	Passive Surveillance	Community	General population	4/1/2020
32298246	Community Transmission of SARS-CoV-2 at Two Family Gatherings — Chicago, Illinois, February–March 2020	4/17/2020	Ghinai	B	MMWR	Descriptive	Case series or cluster	Field Data	Social gathering	General population	3/20/2020
32298247	Characteristics of Health Care Personnel with COVID-19 — United States, February 12–April 9, 2020	4/17/2020	CDCC19RT	A	MMWR	Descriptive	Case series or cluster	Passive Surveillance	Community	Healthcare Workers or First Responders	4/9/2020
32298249	Transmission of COVID-19 to Health Care Personnel During Exposures to a Hospitalized Patient — Solano County, California, February 2020	4/17/2020	Heinzerling	B	MMWR	Descriptive	Case series or cluster	Field Data	Social gathering	Healthcare Workers or First Responders	2/26/2020
32298250	Geographic Differences in COVID-19 Cases, Deaths, and Incidence — United States, February 12–April 7, 2020	4/17/2020	CDCC19RT	A	MMWR	Descriptive	Incidence	Passive Surveillance	Community	General population	4/7/2020
32298251	Hospitalization Rates and Characteristics of Patients Hospitalized with Laboratory-Confirmed Coronavirus Disease 2019 — COVID-NET, 14 States, March 1–30, 2020	4/17/2020	Garg	B	MMWR	Descriptive	Incidence	Active surveillance	Community	General population	3/30/2020
32301962	Symptom Screening at Illness Onset of Health Care Personnel With SARS-CoV-2 Infection in King County, Washington	5/6/2020	Chow	B	JAMA	Descriptive	Case series or cluster	Survey	Healthcare facility	Healthcare Workers or First Responders	3/13/2020
32329971	Presymptomatic SARS-CoV-2 Infections and Transmission in a Skilled Nursing Facility	4/24/2020	Arons	B	NEJM	Descriptive	Case series or cluster	Field Data	Skilled nursing facility	LCTF residents and staff	3/20/2020
32329971	Presymptomatic SARS-CoV-2 Infections and Transmission in a Skilled Nursing Facility	4/24/2020	Aarons	B	NEJM	Descriptive	Case series or clust	Field Data	Skilled nursing f	LCTF residents and	4/3/2020
32352954	COVID-19 Outbreak Among Three Affiliated Homeless Service Sites — King County, Washington, 2020	5/1/2020	Tobolowsky	B	MMWR	Descriptive	Case series or cluster	Field Data	Homeless Facility	Homeless Individuals or Facility Staff	4/8/2020
32352957	Assessment of SARS-CoV-2 Infection Prevalence in Homeless Shelters — Four U.S. Cities, March 27–April 15, 2020	5/1/2020	Mosites	B	MMWR	Descriptive	Case series or cluster	Field Data	Homeless Facility	Homeless Individuals or Facility Staff	4/15/2020
32379729	Characteristics and Clinical Outcomes of Adult Patients Hospitalized with COVID-19 — Georgia, March 2020	5/8/2020	Gold	B	MMWR	Analytic	Cross-sectional	Medical records	Community	General population	3/30/2020
32379731	COVID-19 Among Workers in Meat and Poultry Processing Facilities - 19 States, April 2020.	5/8/2020	Dyal	B	MMWR	Descriptive	Case series or cluster	Passive Surveillance	Other Workplace	Other Occupations	4/27/2020
32382743	COVID-19 Testing, Epidemic Features, Hospital Outcomes, and Household Prevalence, New York State-March 2020	5/8/2020	Rosenberg	C	Clin Infect Dis	Analytic	Retrospective	Passive Surveillance	Community	General population	3/30/2020
32407300	COVID-19 in Correctional and Detention Facilities — United States, February–April 2020	5/15/2020	Wallace	B	MMWR	Descriptive	Incidence	Passive Surveillance	Correctional or detention facility	Prison inmates and staff	4/21/2020
32407301	Public Health Response to COVID-19 Cases in Correctional and Detention Facilities — Louisiana, March–April 2020	5/15/2020	Wallace	B	MMWR	Descriptive	Case series or cluster	Survey	Correctional or detention facility	LCTF residents and staff	4/22/2020
32407303	High SARS-CoV-2 Attack Rate Following Exposure at a Choir Practice — Skagit County, Washington, March 2020	5/15/2020	Hamner	C	MMWR	Descriptive	Case series or cluster	Field Data	Social gathering	General population	4/10/2020
32407306	Preliminary Estimate of Excess Mortality During the COVID-19 Outbreak — New York City, March 11–May 2, 2020	5/15/2020	New York City Department of Health and Mental Hygiene	B	MMWR	Descriptive	Incidence	Vital statistics	Community	General population	5/2/2020
32421144	Seroprevalence of SARS-CoV-2-Specific Antibodies Among Adults in Los Angeles County, California, on April 10-11, 2020	5/18/2020	Sood	C	JAMA	Analytic	Cross-sectional	Field Data	Community	General population	4/14/2020
32437338	High COVID-19 Attack Rate Among Attendees at Events at a Church — Arkansas, March 2020	5/22/2020	James	B	MMWR	Descriptive	Case series or cluster	Field Data	Social gathering	General population	3/16/2020
32437547	Outbreak Investigation of COVID-19 Among Residents and Staff of an Independent and Assisted Living Community for Older Adults in Seattle, Washington	5/20/2020	Roxby	B	JAMA	Descriptive	Case series or cluster	Field Data	Assisted living facility	LCTF residents and staff	5/30/2020
32470316	Investigation and Serologic Follow-Up of Contacts of an Early Confirmed Case-Patient with COVID-19, Washington, USA	5/29/2020	Chu	B	EID	Descriptive	Case series or cluster	Field Data	Community	General population	1/20/2020
32497031	COVID-19 Monitoring and Response Among U.S. Air Force Basic Military Trainees — Texas, March–April 2020	6/5/2020	Marcus	A	MMWR	Descriptive	Incidence	Passive Surveillance	Military facility	Armed Forces	4/18/2020
32525850	SARS-CoV-2 Infections and Serologic Responses from a Sample of U.S. Navy Service Members — USS Theodore Roosevelt, April 2020	6/12/2020	Payne	A	MMWR	Analytic	Cross-sectional	Serosurvey	Military facility	Armed Forces	4/30/2020
32525856	Impact of the COVID-19 Pandemic on Emergency Department Visits — United States, January 1, 2019–May 30, 2020	6/12/2020	Hartnett	A	MMWR	Descriptive	Incidence	Active surveillance	Community	General population	5/30/2020
32548628	Asymptomatic SARS-CoV-2 Infection and COVID-19 Mortality During an Outbreak Investigation in a Skilled Nursing Facility	12/31/2020	Patel	C	Clin Infect Dis	Analytic	Prospective	Field Data	Skilled nursing facility	LCTF residents and staff	3/15/2020
32555134	Coronavirus Disease 2019 Case Surveillance — United States, January 22–May 30, 2020	6/19/2020	Stokes	A	MMWR	Descriptive	Incidence	Passive Surveillance	Community	General population	5/30/2020
32556327	Severe Acute Respiratory Syndrome Coronavirus 2 Infections in Children: Multicenter Surveillance, United States, January-March 2020	11/10/2020	Rha	A	J Pediatric Infect Dis Soc	Descriptive	Incidence	Active surveillance	Community	Children and Adolescents	3/30/2020
32584795	Characteristics of Women of Reproductive Age with Laboratory-Confirmed SARS-CoV-2 Infection by Pregnancy Status — United States, January 22–June 7, 2020	6/26/2020	Ellington	A	MMWR	Analytic	Cross-sectional	Passive Surveillance	Community	Pregnant women	6/7/2020
32584797	Characteristics Associated with Hospitalization Among Patients with COVID-19 — Metropolitan Atlanta, Georgia, March – April 2020	6/26/2020	Killerby	B	MMWR	Analytic	Cross-sectional	Medical records	Community	General population	4/7/2020
32597750	Antibody Responses after Classroom Exposure to Teacher with Coronavirus Disease, March 2020.	6/29/2020	Brown	B	EID	Descriptive	Case series or cluster	Field Data	Primary or secondary school	Children and Adolescents	3/1/2020
32614768	High Proportion of Asymptomatic SARS-CoV-2 Infections in 9 Long-Term Care Facilities, Pasadena, California, USA, April 2020	10/26/2020	Feaster	C	EID	Descriptive	Case series or cluster	Active surveillance	Skilled nursing facility	LCTF residents and staff	4/30/2020
32614809	Exposures Before Issuance of Stay-at-Home Orders Among Persons with Laboratory-Confirmed COVID-19 — Colorado, March 2020	7/3/2020	Marshall	B	MMWR	Descriptive	Case series or cluster	Passive Surveillance	Community	General population	3/26/2020
32614810	Characteristics of Adult Outpatients and Inpatients with COVID-19 — 11 Academic Medical Centers, United States, March–May 2020	7/3/2020	Tenforde	B	MMWR	Analytic	Cross-sectional	Survey	Community	General population	5/24/2020
32614814	COVID-19 Outbreak Among College Students After a Spring Break Trip to Mexico — Austin, Texas, March 26–April 5, 2020	7/3/2020	Lewis	C	MMWR	Descriptive	Case series or cluster	Field Data	Community	College students	4/5/2020
32614815	Screening for SARS-CoV-2 Infection Within a Psychiatric Hospital and Considerations for Limiting Transmission Within Residential Psychiatric Facilities — Wyoming, 2020	7/3/2020	Callaghan	B	MMWR	Descriptive	Case series or cluster	Field Data	Skilled nursing facility	Prison inmates and staff	5/1/2020
32614816	Serial Laboratory Testing for SARS-CoV-2 Infection Among Incarcerated and Detained Persons in a Correctional and Detention Facility — Louisiana, April–May 2020	7/3/2020	Njuguna	B	MMWR	Descriptive	Case series or cluster	Field Data	Correctional or detention facility	Prison inmates and staff	5/21/2020
32620182	Severe Acute Respiratory Syndrome Coronavirus 2 Prevalence, Seroprevalence, and Exposure among Evacuees from Wuhan, China, 2020	7/3/2020	Hallowell	B	EID	Descriptive	Case series or cluster	Field Data	Community	General population	1/28/2020
32628750	Seroprevalence of SARS-CoV-2 Among Frontline Healthcare Personnel During the First Month of Caring for Patients With COVID-19—Nashville, Tennessee	6/6/2020	Stubblefield	A	Clin Infect Dis	Analytic	Cross-sectional	Serosurvey	Healthcare facility	Healthcare Workers or First Responders	4/13/2020
32639228	Coronavirus Disease among Persons with Sickle Cell Disease, United States, March 20–May 21, 2020	7/22/2020	Panepinto	A	EID	Descriptive	Case series or cluster	Passive Surveillance	Community	General population	5/21/2020
32644981	Race/Ethnicity, Underlying Medical Conditions, Homelessness, and Hospitalization Status of Adult Patients with COVID-19 at an Urban Safety-Net Medical Center — Boston, Massachusetts, 2020	7/10/2020	Hsu	A	MMWR	Descriptive	Case series or cluster	Medical records	Community	Homeless Individuals or Facility Staff	5/18/2020
32644985	Initial and Repeated Point Prevalence Surveys to Inform SARS-CoV-2 Infection Prevention in 26 Skilled Nursing Facilities — Detroit, Michigan, March–May 2020	7/10/2020	Sanchez	B	MMWR	Descriptive	Incidence	Field Data	Skilled nursing facility	LCTF residents and staff	5/8/2020
32644986	Update: COVID-19 Among Workers in Meat and Poultry Processing Facilities — United States, April–May 2020	7/10/2020	Waltenburg	B	MMWR	Descriptive	Incidence	Passive Surveillance	Community	Other Occupations	5/31/2020
32648546	Cumulative incidence and diagnosis of SARS-CoV-2 infection in New York	6/17/2020	Rosenberg	C	Ann Epidemiology	Analytic	Cross-sectional	Serosurvey	Community	General population	3/29/2020
32673296	Symptom Profiles of a Convenience Sample of Patients with COVID-19 — United States, January–April 2020	7/17/2020	Burke	B	MMWR	Descriptive	Case series or cluster	Passive Surveillance	Community	General population	6/3/2020
32673298	Characteristics of Persons Who Died with COVID-19 — United States, February 12–May 18, 2020	7/17/2020	Wortham	B	MMWR	Descriptive	Case series or cluster	Passive Surveillance	Community	General population	5/18/2020
32673300	Absence of Apparent Transmission of SARS-CoV-2 from Two Stylists After Exposure at a Hair Salon with a Universal Face Covering Policy — Springfield, Missouri, May 2020	7/17/2020	Hendrix	C	MMWR	Descriptive	Case series or cluster	Field Data	Community	General population	5/15/2020
32678072	Detection and Genetic Characterization of Community-Based SARS-CoV-2 Infections — New York City, March 2020	7/17/2020	Bushman	B	MMWR	Descriptive	Case series or cluster	Active surveillance	Community	General population	3/20/2020
32701938	Population Point Prevalence of SARS-CoV-2 Infection Based on a Statewide Random Sample — Indiana, April 25–29, 2020	7/24/2020	Menachemi	C	MMWR	Analytic	Cross-sectional	Field Data	Community	General population	4/29/2020
32701941	Estimated Community Seroprevalence of SARS-CoV-2 Antibodies — Two Georgia Counties, April 28–May 3, 2020	7/24/2020	Biggs	B	MMWR	Analytic	Cross-sectional	Serosurvey	Community	General population	5/3/2020
32723697	Assessing racial and ethnic disparities using a COVID-19 outcomes continuum for New York State	6/20/2020	Holtgrave	C	Ann Epidemiol	Analytic	Case series or cluster	Passive Surveillance	Community	General population	3/29/2020
32730236	Notes from the Field: Public Health Efforts to Mitigate COVID-19 Transmission During the April 7, 2020 Election — City of Milwaukee, Wisconsin, March 13–May 5, 2020	7/31/2020	Paradis	B	MMWR	Descriptive	Incidence	Passive Surveillance	Community	General population	5/5/2020
32744615	Coronavirus Disease 2019 Outbreak in a San Francisco Homeless Shelter	8/3/2020	Imbert	C	Clin Infect Dis	Descriptive	Case series or cluster	Field Data	Homeless Facility	Homeless Individuals or Facility Staff	4/12/2020
32755020	Exposures in adult outpatients with COVID-19 infection during early community transmission, Tennessee	8/4/2020	Tenforde	A	Influenza Other Respir Viruses	Descriptive	Case series or cluster	Survey	Community	General population	4/11/2020
32759914	COVID-19 Outbreak Among Employees at a Meat Processing Facility — South Dakota, March–April 2020	8/7/2020	Steinberg	B	MMWR	Descriptive	Case series or cluster	Field Data	Other Workplace	Other Occupations	5/25/2020
32759918	Characteristics and Outcomes of Contacts of COVID-19 Patients Monitored Using an Automated Symptom Monitoring Tool — Maine, May–June 2020	8/7/2020	Krueger	B	MMWR	Descriptive	Case series or cluster	Field Data	Community	General population	6/26/2020

PMID	SAR	SerialInterval	GrowthRate	InfectionPredictor	IpAgeOrSex	IpRaceOrEthnicity	IpClinical	IpBehavioral	IpOccupational	IpEnvironmental	IpResidential	IpReinfection	IpGeospatial	SeverityPredictor	SpAgeOrSex	SpRaceOrEthnicity	SpClinical	SpBehavioral	SpOccupational	SpReinfection	SpVariant	SpGeospatial	SymptomaticFraction	InfectionCaseRatio	CaseHospitalizationRatio	CaseFatalityRatio	InfectionHospitalizationRatio	HospitalFatalityRatio	InfectionFatalityRatio	IncidenceMeasure	ImInfection	ImCase	ImEDvisit	ImHospitalization	ImMortality	ImSeroprevalence	ImExcessDeath
32004427																																					
32027631																																					
32053579																																					
32106216																																					
32134909	×																																				
32178768																																					
32214079																																					
32214083																																					
32220208																																					
32232421																														×							
32240123																																					
32240128																																					
32240285																																					
32271724																																					
32271726																																					
32271728																																					
32298245																														×		×					
32298246																																					
32298247																																					
32298249																																					
32298250																														×		×					
32298251																														×				×			
32301962																																					
32329971																																					
32329971																																					
32352954																																					
32352957																														×							
32379729														×			×																				
32379731																																					
32382743	×			×	×						×																										
32407300																														×		×		×	×		
32407301																																					
32407303																																					
32407306																														×		×			×		×
32421144				X	X	X																								X						X	
32437338																																					
32437547																																					
32470316																																					
32497031																														×		×					
32525850				×	×			×															×														
32525856																														×			×				
32548628																							×			×											
32555134																														×		×					
32556327																														×		×					
32584795														×			×																				
32584797														×	×	×	×																				
32597750																																					
32614768																																					
32614809																																					
32614810														×	×	×	×																				
32614814																																					
32614815																																					
32614816																																					
32620182																																					
32628750				×	×		×		×																					×						×	
32639228																																					
32644981																																					
32644985																														×		×					
32644986																														×		×					
32648546				×	×	×																								×						×	
32673296																																					
32673298																																					
32673300																																					
32678072																																					
32701938				×	×	×							×										×							×		×				×	
32701941				×	×		×		×		×																			×						×	
32723697														×		×													×								
32730236																														×		×		×			
32744615																																					
32755020																																					
32759914																																					
32759918																																					

32759920	Notes from the Field: Characteristics of Meat Processing Facility Workers with Confirmed SARS-CoV-2 Infection — Nebraska, April–May 2020	8/7/2020	Donahue	B	MMWR	Descriptive	Case series or cluster	Field Data	Other Workplace	Other Occupations	5/25/2020																																					
32759921	SARS-CoV-2 Transmission and Infection Among Attendees of an Overnight Camp — Georgia, June 2020	8/7/2020	Szablewski	B	MMWR	Descriptive	Case series or cluster	Field Data	Social gathering	Children and Adolescents	6/25/2020																																					
32790655	Facility-Wide Testing for SARS-CoV-2 in Nursing Homes — Seven U.S. Jurisdictions, March–June 2020	8/14/2020	Hatfield	B	MMWR	Descriptive	Incidence	Field Data	Skilled nursing facility	LCTF residents and staff	6/14/2020																													×		×					
32790658	Notes from the Field: Seroprevalence Estimates of SARS-CoV-2 Infection in Convenience Sample — Oregon, May 11–June 15, 2020	8/14/2020	Sutton	C	MMWR	Analytic	Cross-sectional	Serosurvey	Community	General population	6/25/2020				×	×																									×						×	
32790661	Transmission of SARS-CoV-2 Involving Residents Receiving Dialysis in a Nursing Home — Maryland, April 2020	8/14/2020	Bigelow	B	MMWR	Descriptive	Case series or cluster	Field Data	Community	LCTF residents and staff	4/30/2020																																					
32790664	Risk Factors for Intensive Care Unit Admission and In-hospital Mortality Among Hospitalized Adults Identified through the US Coronavirus Disease 2019 (COVID-19)-Associated Hospitalization Surveillance Network (COVID-NET)	7/16/2020	Kim	B	Clin Infect Dis	Analytic	Retrospective	Active surveillance	Community	General population	5/2/2020														×	×		×																				
32790860	Point Prevalence Testing of Residents for SARS-CoV-2 in a Subset of Connecticut Nursing Homes	8/10/2020	Parikh	B	JAMA	Descriptive	Case series or cluster	Field Data	Skilled nursing facility	LCTF residents and staff	5/19/2020																																					
32803237	Coronavirus Disease 2019 (COVID-19) Infections Among Healthcare Workers, Los Angeles County, February-May 2020	8/17/2020	Hartmann	C	Clin Infect Dis	Descriptive	Case series or cluster	Passive Surveillance	Healthcare facility	Healthcare Workers or First Responders	5/31/2020																																					
32817597	Mass Testing for SARS-CoV-2 in 16 Prisons and Jails — Six Jurisdictions, United States, April–May 2020	8/21/2020	Hagan	B	MMWR	Descriptive	Incidence	Active surveillance	Correctional or detention facility	Prison inmates and staff	5/20/2020																													×		×					
32817601	Notes from the Field: COVID-19 Prevention Practices in State Prisons — Puerto Rico, 2020	8/21/2020	Davalantes	B	MMWR	Descriptive	Case series or cluster	Passive Surveillance	Correctional or detention facility	Prison inmates and staff	8/17/2020																																					
32817602	Disparities in Incidence of COVID-19 Among Underrepresented Racial/Ethnic Groups in Counties Identified as Hotspots During June 5–18, 2020 — 22 States, February–June 2020	8/21/2020	Moore	B	MMWR	Analytic	Ecologic	Passive Surveillance	Community	General population	6/18/2020				×									×																	×		×					
32817604	Racial and Ethnic Disparities Among COVID-19 Cases in Workplace Outbreaks by Industry Sector — Utah, March 6–June 5, 2020	8/21/2020	Bui	B	MMWR	Analytic	Ecologic	Passive Surveillance	Other Workplace	Other Occupations	6/5/2020				×	×	×																															
32817606	Trends in Number and Distribution of COVID-19 Hotspot Counties — United States, March 8–July 15, 2020	8/21/2020	Oster	B	MMWR	Descriptive	Incidence	Passive Surveillance	Community	General population	7/15/2020																													×		×					
32853185	Limited Secondary Transmission of SARS-CoV-2 in Child Care Programs — Rhode Island, June 1–July 31, 2020	8/28/2020	Link-Gelles	B	MMWR	Descriptive	Case series or cluster	Active surveillance	Childcare facility	General population	7/31/2020																																					
32853190	Notes from the Field: Universal Statewide Laboratory Testing for SARS-CoV-2 in Nursing Homes — West Virginia, April 21–May 8, 2020	8/28/2020	McBee	B	MMWR	Descriptive	Case series or cluster	Active surveillance	Skilled nursing facility	LCTF residents and staff	5/8/2020																													×							
32853193	COVID-19 Among American Indian and Alaska Native Persons — 23 States, January 31–July 3, 2020	8/28/2020	Hatcher	B	MMWR	Descriptive	Incidence	Passive Surveillance	Community	General population	7/3/2020																													×		×					
32875494	Cross-Sectional Prevalence of SARS-CoV-2 Among Skilled Nursing Facility Employees and Residents Across Facilities in Seattle	9/1/2020	Weil	C	J Gen Intern Med	Descriptive	Incidence	Field Data	Skilled nursing facility	LCTF residents and staff	5/13/2020																													×		×					
32877446	Enhanced contact investigations for nine early travel-related cases of SARS-CoV-2 in the United States	9/2/2020	Burke	B	Plos One	Analytic	Case series or cluster	Field Data	Community	General population	2/15/2020	X																																				
32881855	Seroprevalence of SARS-CoV-2 Among Frontline Health Care Personnel in a Multistate Hospital Network — 13 Academic Medical Centers, April–June 2020	9/4/2020	Self	A	MMWR	Analytic	Cross-sectional	Serosurvey	Healthcare facility	Healthcare Workers or First Responders	6/30/2020				×	×	×	×		×																					×						×	
32898272	Prevalence among People Experiencing Homelessness and Homelessness Service Staff during Early Community Transmission in Atlanta, Georgia, April–May 2020	9/8/2020	Yoon	A	Clin Infect Dis	Analytic	Cross-sectional	Field Data	Homeless Facility	Homeless Individuals or Facility Staff	5/6/2020				×							×																										
32915165	Community and Close Contact Exposures Associated with COVID-19 Among Symptomatic Adults ≥18 Years in 11 Outpatient Health Care Facilities — United States, July 2020	9/11/2020	Fisher	A	MMWR	Analytic	Case control	Survey	Community	General population	7/29/2020				×	×			×																													
32941409	Association Between CMS Quality Ratings and COVID-19 Outbreaks in Nursing Homes — West Virginia, March 17–June 11, 2020	9/18/2020	Bui	B	MMWR	Analytic	Ecologic	Passive Surveillance	Skilled nursing facility	LCTF residents and staff	6/11/2020				×							×																										
32941413	Preventing COVID-19 Outbreaks in Long-term Care Facilities Through Preemptive Testing of Residents and Staff Members — Fulton County, Georgia, March–May 2020	9/18/2020	Telford	C	MMWR	Analytic	Ecologic	Field Data	Skilled nursing facility	LCTF residents and staff	5/18/2020				×							×																										
32941417	SARS-CoV-2–Associated Deaths Among Persons Aged <21 Years — United States, February 12–July 31, 2020	9/18/2020	Bixler	B	MMWR	Descriptive	Incidence	Passive Surveillance	Community	Children and Adolescents	7/31/2020																													×		×			×		
32941418	Transmission Dynamics of COVID-19 Outbreaks Associated with Child Care Facilities — Salt Lake City, Utah, April–July 2020	9/18/2020	Lopez	B	MMWR	Descriptive	Case series or cluster	Field Data	Childcare facility	General population	7/10/2020																																					
32945846	Risk Factors for Coronavirus Disease 2019 (COVID-19)-Associated Hospitalization: COVID-19-Associated Hospitalization Surveillance Network and Behavioral Risk Factor Surveillance System	9/18/2020	Ko	B	Clin Infect Dis	Analytic	Cross-sectional	Active surveillance	Community	General population	6/23/2020														X	X	X	X																				
32956614	SARS-CoV-2 Seroprevalence among Healthcare, First Response, and Public Safety Personnel, Detroit Metropolitan Area, Michigan, USA, May-June 2020	9/21/2020	Akinbami	A	EID	Analytic	Cross-sectional	Serosurvey	Healthcare facility	Healthcare Workers or First Responders	6/13/2020				×	×	×		×	×				×																	×						×	
32966272	Serial Testing for SARS-CoV-2 and Virus Whole Genome Sequencing Inform Infection Risk at Two Skilled Nursing Facilities with COVID-19 Outbreaks — Minnesota, April–June 2020	9/18/2020	Taylor	B	MMWR	Descriptive	Case series or cluster	Field Data	Skilled nursing facility	LCTF residents and staff	7/21/2020																																					
32970655	Characteristics and Maternal and Birth Outcomes of Hospitalized Pregnant Women with Laboratory-Confirmed COVID-19 — COVID-NET, 13 States, March 1–August 22, 2020	9/25/2020	Delahoy	B	MMWR	Descriptive	Case series or cluster	Active surveillance	Community	Pregnant women	8/22/2020																																					
32970656	Disparities in COVID-19 Incidence, Hospitalizations, and Testing, by Area-Level Deprivation — Utah, March 3–July 9, 2020	9/25/2020	Lewis	B	MMWR	Analytic	Ecologic	Passive Surveillance	Community	General population	7/9/2020				×									×																								
32970660	SARS-CoV-2 Infection Among Hospitalized Pregnant Women: Reasons for Admission and Pregnancy Characteristics — Eight U.S. Health Care Centers, March 1–May 30, 2020	9/25/2020	Panagiotakopoulos	A	MMWR	Descriptive	Case series or cluster	Medical records	Community	Pregnant women	5/30/2020																																					
32970661	Update: Characteristics of Health Care Personnel with COVID-19 — United States, February 12–July 16, 2020	9/25/2020	Hughes	B	MMWR	Descriptive	Case series or cluster	Passive Surveillance	Community	Healthcare Workers or First Responders	7/16/2020																																					
33001869	COVID-19 Trends Among School-Aged Children — United States, March 1–September 19, 2020	10/2/2020	Leeb	A	MMWR	Descriptive	Incidence	Passive Surveillance	Community	Children and Adolescents	9/19/2020																													×		×					
33001871	Multiple COVID-19 Clusters on a University Campus — North Carolina, August 2020	10/2/2020	Wilson	B	MMWR	Descriptive	Case series or cluster	Passive Surveillance	College or university	General population	8/25/2020																																					
33001872	Changing Age Distribution of the COVID-19 Pandemic — United States, May–August 2020	10/2/2020	Boehmer	A	MMWR	Descriptive	Incidence	Passive Surveillance	Community	General population	8/30/2020																													×		×	×				
33006586	Recent Increase in COVID-19 Cases Reported Among Adults Aged 18–22 Years — United States, May 31–September 5, 2020	10/2/2020	Salvatore	A	MMWR	Descriptive	Incidence	Passive Surveillance	Community	General population	9/5/2020																													×		×					
33031365	Adolescent with COVID-19 as the Source of an Outbreak at a 3-Week Family Gathering — Four States, June–July 2020	10/9/2020	Schwartz	B	MMWR	Descriptive	Case series or cluster	Field Data	Social gathering	General population	8/30/2020																																					
33031366	Trends in COVID-19 Incidence After Implementation of Mitigation Measures — Arizona, January 22–August 7, 2020	10/9/2020	Gallaway	B	MMWR	Descriptive	Incidence	Passive Surveillance	Community	General population	8/7/2020																													×		×					
33033178	Symptoms and Transmission of SARS-CoV-2 Among Children - Utah and Wisconsin, March-May 2020	10/8/2020	Laws	B	Pediatrics	Analytic	Prospective	Field Data	Community	General population	5/30/2020	×			×	X																																
33044323	COVID-19 Susceptibility and Outcomes Among People Living With HIV in San Francisco	11/20/2020	Sachdev	C	J Acquir Immune Defic Syndr	Analytic	Cross-sectional	Passive Surveillance	Community	General population	9/30/2020				X			X																														
33056949	Transmission Dynamics by Age Group in COVID-19 Hotspot Counties — United States, April–September 2020	10/16/2020	Oster	A	MMWR	Descriptive	Incidence	Passive Surveillance	Community	General population	7/31/2020																													×		×					
33056952	An Outbreak of COVID-19 Associated with a Recreational Hockey Game — Florida, June 2020	10/16/2020	Atrubin	C	MMWR	Descriptive	Case series or cluster	Field Data	Social gathering	General population	6/21/2020																																					
33056953	Factors Influencing Risk for COVID-19 Exposure Among Young Adults Aged 18–23 Years — Winnebago County, Wisconsin, March–July 2020	10/16/2020	Wilson	B	MMWR	Descriptive	Case series or cluster	Passive Surveillance	Community	General population	7/18/2020																																					
33058538	Characterization of community-wide transmission of SARS-CoV-2 in congregate living settings and local public health-coordinated response during the initial phase of the COVID-19 pandemic	10/15/2020	Terabuh	C	Influenza Other Respir Viruses	Descriptive	Case series or cluster	Field Data	Congregate living facilities (multiple)	General population	5/15/2020																																					
33075274	Coronavirus Disease among Workers in Food Processing, Food Manufacturing, and Agriculture Workplaces	10/19/2020	Waltenburg	B	EID	Descriptive	Incidence	Passive Surveillance	Other Workplace	Other Occupations	5/31/2020																													×		×					
33077014	Healthcare-associated outbreak of severe acute respiratory coronavirus virus 2 (SARS-CoV-2) in a rural hospital in Missouri.	10/20/2020	Turabelidze	C	Infect Control Hosp Epidemiol	Descriptive	Case series or cluster	Field Data	Healthcare facility	Healthcare Workers or First Responders	3/11/2020																																					
33090977	Association Between Social Vulnerability and a County's Risk for Becoming a COVID-19 Hotspot — United States, June 1–July 25, 2020.	10/23/2020	Dasgupta	A	MMWR	Analytic	Ecologic	Passive Surveillance	Community	General population	7/25/2020				×									×																								
33090978	Excess Deaths Associated with COVID-19, by Age and Race and Ethnicity — United States, January 26–October 3, 2020.	10/23/2020	Rossen	A	MMWR	Analytic	Ecologic	Vital statistics	Community	General population	10/3/2020																																					×
33090983	Mitigating a COVID-19 Outbreak Among Major League Baseball Players — United States, 2020.	10/23/2020	Murray	B	MMWR	Descriptive	Case series or cluster	Field Data	Community	Other Occupations	7/24/2020																																					
33090984	Race, Ethnicity, and Age Trends in Persons Who Died from COVID-19 — United States, May–August 2020.	10/23/2020	Gold	A	MMWR	Descriptive	Incidence	Vital statistics	Community	General population	8/31/2020																													×					×		
33090987	Risk for In-Hospital Complications Associated with COVID-19 and Influenza — Veterans Health Administration, United States, October 1, 2018–May 31, 2020.	10/23/2020	Cates	A	MMWR	Analytic	Retrospective	Medical records	Community	General population	5/31/2020														×			×																				
33108976	Lack of Serologic Evidence of Infection Among Health Care Personnel and Other Contacts of First 2 Confirmed Patients With COVID-19 in Illinois, 2020	10/27/2020	McPherson	B	Public Health Rep	Descriptive	Case series or cluster	Field Data	Healthcare facility	Healthcare Workers or First Responders	1/17/2020																																					
33119554	COVID-19–Associated Hospitalizations Among Health Care Personnel — COVID-NET, 13 States, March 1–May 31, 2020.	10/30/2020	Kambhampati	B	MMWR	Descriptive	Case series or cluster	Active surveillance	Healthcare facility	Healthcare Workers or First Responders	5/31/2020																																					
33119557	SARS-CoV-2 Exposure and Infection Among Health Care Personnel — Minnesota, March 6–July 11, 2020	10/30/2020	Fell	B	MMWR	Analytic	Cross-sectional	Field Data	Healthcare facility	Healthcare Workers or First Responders	7/11/2020				×					×																												
33119558	COVID-19 Outbreak at an Overnight Summer School Retreat — Wisconsin, July–August 2020	10/30/2020	Pray	B	MMWR	Descriptive	Case series or cluster	Field Data	Social gathering	Children and Adolescents	8/11/2020																																					
33119564	COVID-19 in a Correctional Facility Employee Following Multiple Brief Exposures to Persons with COVID-19 — Vermont, July–August 2020	10/30/2020	Pringle	B	MMWR	Descriptive	Case series or cluster	Field Data	Correctional or detention facility	Prison inmates and staff	8/11/2020																																					
33124003	An Ecologic Study of Disparities in COVID-19 Incidence and Case Fatality in Oakland County, MI, USA, During a State-Mandated Shutdown	10/29/2020	Akanbi	C	J Racial Ethn Health Disparities	Analytic	Ecologic	Passive Surveillance	Community	General population	5/16/2020				X									X	X							X																
33151915	Network Characteristics and Visualization of COVID-19 Outbreak in a Large Detention Facility in the United States - Cook County, Illinois, 2020	11/6/2020	Kırbıyık	B	MMWR	Analytic	Cross-sectional	Field Data	Correctional or detention facility	Prison inmates and staff	4/30/2020				×							×																										
33151916	Transmission of SARS-COV-2 Infections in Households — Tennessee and Wisconsin, April–September 2020	11/6/2020	Grijalva	A	MMWR	Analytic	Prospective	Field Data	Community	General population	9/30/2020	×			×	×	×					×																										
33151917	Birth and Infant Outcomes Following Laboratory-Confirmed SARS-CoV-2 Infection in Pregnancy — SET-NET, 16 Jurisdictions, March 29–October 14, 2020	11/6/2020	Woodworth	B	MMWR	Descriptive	Case series or cluster	Active surveillance	Community	General population	10/14/2020																																					
33151921	Characteristics of Symptomatic Women of Reproductive Age with Laboratory-Confirmed SARS-CoV-2 Infection by Pregnancy Status — United States, January 22–October 3, 2020.	11/6/2020	Zambrano	A	MMWR	Analytic	Retrospective	Passive Surveillance	Community	General population	10/3/2020														×			×																				
33151922	Outbreak Illustrating the Challenges in Limiting the Spread of the Virus — Hopi Tribe, May–June 2020.	11/6/2020	Hirschman	B	MMWR	Descriptive	Case series or cluster	Field Data	Community	General population	6/22/2020																																					
33166098	COVID-19 Transmission in a Psychiatric Long-Term Care Rehabilitation Facility: An Observational Study	11/5/2020	Xiong	C	Prim Care Companion CNS Disord	Descriptive	Case series or cluster	Field Data	Healthcare facility	LCTF residents and staff	4/24/2020																																					
33180752	Multiple COVID-19 Outbreaks Linked to a Wedding Reception in Rural Maine — August 7–September 14, 2020.	11/13/2020	Mahale	B	MMWR	Descriptive	Case series or cluster	Field Data	Community	General population	9/14/2020																																					
33180753	COVID-19 Outbreak in an Amish Community - Ohio, May 2020	11/13/2020	Ali	B	MMWR	Descriptive	Case series or cluster	Field Data	Community	General population	5/20/2020																																					
33180757	Declines in SARS-CoV-2 Transmission, Hospitalizations, and Mortality After Implementation of Mitigation Measures— Delaware, March–June 2020.	11/13/2020	Kanu	B	MMWR	Descriptive	Incidence	Passive Surveillance	Community	General population	6/25/2020																													×		×		×	×		
33185244	Household Transmission of SARS-CoV-2 in the United States	8/16/2020	Lewis	B	Clin Infect Dis	Analytic	Prospective	Field Data	Community	General population	4/25/2020	×			×			×																														
33210336	Estimation of differential occupational risk of COVID-19 by comparing risk factors with case data by occupational group	11/18/2020	Zhang	C	Am J Ind Med	Analytic	Ecologic	Passive Surveillance	Community	General population	6/16/2020				×					×																												
33211679	Characterization of COVID-19 in Assisted Living Facilities — 39 States, October 2020.	11/20/2020	Yi	A	MMWR	Descriptive	Incidence	Passive Surveillance	Assisted living facility	LCTF residents and staff	10/15/2020																													×		×			×		
33211680	COVID-19 Outbreak — New York City, February 29–June 1, 2020.	11/20/2020	Thompson	C	MMWR	Descriptive	Incidence	Passive Surveillance	Community	General population	6/1/2020																													×		×					
33211682	COVID-19 Stats: COVID-19 Incidence, by Urban-Rural Classification — United States, January 22–October 31, 2020.	11/20/2020	Duca	A	MMWR	Descriptive	Incidence	Passive Surveillance	Community	General population	10/31/2020																													×		×					
33237889	Trends in County-Level COVID-19 Incidence in Counties With and Without a Mask Mandate — Kansas, June 1–August 23, 2020.	11/27/2020	Van Dyke	B	MMWR	Descriptive	Incidence	Passive Surveillance	Community	General population	8/23/2020																													×		×					
33237891	COVID-19 Outbreak Associated with a 10-Day Motorcycle Rally in a Neighboring State — Minnesota, August–September 2020.	11/27/2020	Firestone	B	MMWR	Descriptive	Case series or cluster	Field Data	Community	General population	8/30/2020																																					

33248030	Community Coronavirus Disease 2019 Activity Level and Nursing Home Staff Testing for Active Severe Acute Respiratory Syndrome Coronavirus 2 Infection in Indiana	10/28/2020	Blackburn	C	J Am Med Dir Assoc .	Analytic	Ecologic	Field Data	Skilled nursing facility	LCTF residents and staff	6/30/2020		X								X														
33270101	Identification of Presymptomatic and Asymptomatic Cases Using Cohort-Based Testing Approaches at a Large Correctional Facility-Chicago, Illinois, USA, May 2020	12/3/2020	Wadhwa	B	Clin Infect Dis	Analytic	Prospective	Field Data	Correctional or detention facility	Prison inmates and staff	5/19/2020															X									
33270136	Characteristics of Adults Aged 18–49 Years Without Underlying Conditions Hospitalized With Laboratory-Confirmed Coronavirus Disease 2019 in the United States: COVID-NET—March–August 2020	12/3/2020	Owusu	A	Clin Infect Dis	Descriptive	Case series or cluster	Active surveillance	Community	General population	8/1/2020																								
33270609	Multidisciplinary Community-Based Investigation of a COVID-19 Outbreak Among Marshallese and Hispanic/Latino Communities — Benton and Washington Counties, Arkansas, March–June 2020.	12/4/2020	Center	B	MMWR	Descriptive	Case series or cluster	Passive Surveillance	Community	General population	6/13/2020																								
33270613	Disproportionate Incidence of COVID-19 Infection, Hospitalizations, and Deaths Among Persons Identifying as Hispanic or Latino — Denver, Colorado March–October 2020.	12/4/2020	Podewils	C	MMWR	Analytic	Retrospective	Passive Surveillance	Community	General population	10/6/2020		X		X																				
33275874	Characteristics and Timing of Initial Virus Shedding in Severe Acute Respiratory Syndrome Coronavirus 2, Utah, USA.	12/4/2020	Lewis	B	EID	Descriptive	Case series or cluster	Field Data	Community	General population	4/25/2020																								
33301432	COVID-19 Mortality Among American Indian and Alaska Native Persons — 14 States, January–June 2020.	12/11/2020	Arrazola	C	MMWR	Descriptive	Incidence	Passive Surveillance	Community	General population	6/30/2020																		X				X		
33308337	Comparison of symptomatic and asymptomatic infections due to severe acute respiratory coronavirus virus 2 (SARS-CoV-2) in San Francisco long-term care facilities	12/14/2020	Louie	C	Infect Control Hosp Epidemiol	Descriptive	Case series or cluster	Active surveillance	Skilled nursing facility	LCTF residents and staff	6/15/2020																								
33308357	Timely intervention and control of a novel coronavirus (COVID-19) outbreak at a large skilled nursing facility-San Francisco, California, 2020	12/14/2020	Karmarkar	B	Infect Control Hosp Epidemiol	Descriptive	Case series or cluster	Field Data	Skilled nursing facility	LCTF residents and staff	5/4/2020																								
33327990	SARS-CoV-2 Infections among Recent Organ Recipients, March–May 2020, United States	12/16/2020	Jones	B	EID	Descriptive	Case series or cluster	Field Data	Community	General population	5/30/2020																								
33332291	Factors That Might Affect SARS-CoV-2 Transmission Among Foreign-Born and U.S.-Born Poultry Facility Workers - Maryland, May 2020	12/18/2020	Rubenstein	B	MMWR	Analytic	Cross-sectional	Field Data	Other Workplace	Other Occupations	5/30/2020		X		X																				
33332298	Factors Associated with Positive SARS-CoV-2 Test Results in Outpatient Health Facilities and Emergency Departments Among Children and Adolescents Aged <18 Years — Mississippi, September–November 2020.	12/18/2020	Hobbs	B	MMWR	Analytic	Cross-sectional	Survey	Community	Children and Adolescents	11/5/2020		X	X	X																				
33332299	Health Center Testing for SARS-CoV-2 During the COVID-19 Pandemic - United States, June 5-October 2, 2020	12/18/2020	Romero	A	MMWR	Descriptive	Case series or cluster	Survey	Community	General population	10/2/2020																								
33338028	COVID-19-Related Hospitalization Rates and Severe Outcomes Among Veterans From 5 Veterans Affairs Medical Centers: Hospital-Based Surveillance Study	1/22/2021	Cardemil	A	JMIR Public Health Surveill	Analytic	Retrospective	Active surveillance	Healthcare facility	General population	7/17/2020											X	X	X											
33382674	COVID-19 Stats: COVID-19 Incidence, by Age Group — United States, March 1–November 14, 2020	1/1/2021	Duca	A	MMWR	Descriptive	Incidence	Passive Surveillance	Community	General population	11/20/2020																		X		X				
33382676	Implications of Shortened Quarantine Among Household Contacts of Index Patients with Confirmed SARS-CoV-2 Infection — Tennessee and Wisconsin, April–September 2020.	7/20/2021	Rolfes	A	MMWR	Analytic	Prospective	Field Data	Community	General population	9/30/2020	X																							
33395380	Rapid Transmission of Severe Acute Respiratory Syndrome Coronavirus 2 in Detention Facility, Louisiana, USA, May–June, 2020.	1/4/2021	Wallace	B	EID	Descriptive	Case series or cluster	Field Data	Correctional or detention facility	Prison inmates and staff	6/20/2020																								
33411698	Participation in Fraternity and Sorority Activities and the Spread of COVID-19 Among Residential University Communities — Arkansas, August 21–September 5, 2020.	1/8/2021	Vang	B	MMWR	Descriptive	Case series or cluster	Medical records	College or university	College students	9/5/2020																								
33411699	Opening of Large Institutions of Higher Education and County-Level COVID-19 Incidence — United States, July 6–September 17, 2020.	1/8/2021	Leidner	A	MMWR	Descriptive	Incidence	Passive Surveillance	Community	General population	9/17/2020																		X		X				
33411700	Assessment of Day-7 Postexposure Testing of Asymptomatic Contacts of COVID-19 Patients to Evaluate Early Release from Quarantine — Vermont, May–November 2020.	1/8/2021	Jones	C	MMWR	Descriptive	Case series or cluster	Medical records	Community	General population	11/30/2020																								
33417591	Time from Start of Quarantine to SARS-CoV-2 Positive Test Among Quarantined College and University Athletes — 17 States, June–October 2020.	1/8/2021	Atherstone	A	MMWR	Descriptive	Case series or cluster	Serosurvey	College or university	College students	10/31/2020																								
33444301	Rates of COVID-19 Among Residents and Staff Members in Nursing Homes — United States, May 25–November 22, 2020.	1/15/2021	Bagchi	A	MMWR	Descriptive	Incidence	Passive Surveillance	Skilled nursing facility	LCTF residents and staff	11/22/2020																		X		X				
33458740	Population-Based Estimates of Coronavirus Disease 2019 (COVID-19)-like Illness, COVID-19 Illness, and Rates of Case Ascertainment, Hospitalizations, and Deaths-Noninstitutionalized New York City Residents, March-April 2020.	1/18/2021	Alroy	B	Clin Infect Dis	Analytic	Cross-sectional	Survey	Community	General population	4/30/2020																		X	X		X	X		
33476314	COVID-19 Trends Among Persons Aged 0–24 Years — United States, March 1–December 12, 2020	1/22/2021	Leidman	A	MMWR	Descriptive	Incidence	Passive Surveillance	Community	General population	12/12/2020																		X		X	X	X		
33493106	Prevalence of SARS-CoV-2 Antibodies in First Responders and Public Safety Personnel, New York City, New York, USA, May–July 2020.	1/25/2021	Sami	B	EID	Analytic	Case control	Serosurvey	Community	Healthcare Workers or First Responders	7/31/2020		X	X	X	X	X	X		X									X					X	
33496225	Symptom Profiles and Progression in Hospitalized and Nonhospitalized Patients with Coronavirus Disease, Colorado, USA, 2020.	1/5/2021	Vahey	B	EID	Analytic	Cross-sectional	Survey	Community	General population	4/5/2020											X			X										
33496649	SARS-CoV-2 Infection and Mitigation Efforts among Office Workers, Washington, DC, USA.	1/8/2021	Sami	A	EID	Analytic	Cross-sectional	Field Data	Other Workplace	General population	10/31/2020		X	X	X																				
33504612	SARS-CoV-2 Transmission Dynamics in a Sleep-Away Camp	1/27/2021	Szablewski	B	Pediatrics	Descriptive	Case series or cluster	Field Data	Social gathering	Children and Adolescents	8/25/2020																								
33507887	Trends in Outbreak-Associated Cases of COVID-19 — Wisconsin, March–November 2020	1/29/2021	Kocharian	B	MMWR	Descriptive	Case series or clust	Passive Surveillance	Community	General population	11/16/2020																		X						
33507894	Response to a COVID-19 Outbreak on a University Campus — Indiana, August 2020	1/29/2021	Fox	C	MMWR	Descriptive	Case series or cluster	Active surveillance	College or university	College students	10/8/2020																								
33507895	SARS-CoV-2 Transmission Associated with High School Wrestling Tournaments — Florida, December 2020–January 2021	1/29/2021	Atherstone	B	MMWR	Descriptive	Case series or cluster	Field Data	Social gathering	General population	1/7/2021																								
33542329	Estimated seroprevalence of SARS-CoV-2 antibodies among adults in Orange County California.	2/4/2021	Bruckner	C	Scientific Reports	Analytic	Cross-sectional	Serosurvey	Community	General population	8/16/2020		X	X	X														X					X	
33553098	The Relationship Between Social Vulnerability and COVID-19 Incidence Among Louisiana Census Tracts	1/20/2021	Biggs	B	Front Public Health	Analytic	Ecologic	Passive Surveillance	Community	General population	8/24/2020		X								X														
33571176	Decline in COVID-19 Hospitalization Growth Rates Associated with Statewide Mask Mandates — 10 States, March–October 2020	2/12/2021	Joo	A	MMWR	Analytic	Ecologic	Passive Surveillance	Community	General population	10/22/2021																								
33591249	Characteristics of SARS-CoV-2 Transmission among Meat Processing Workers in Nebraska, USA, and Effectiveness of Risk Mitigation Measures.	2/16/2021	Herstein	C	EID	Analytic	Retrospective	Passive Surveillance	Other Workplace	Other Occupations	7/31/2020		X				X		X																
33600249	Seroprevalence of SARS-CoV-2 Antibodies in Rhode Island From a Statewide Random Sample	2/18/2021	Chan	C	AJPH	Analytic	Cross-sectional	Serosurvey	Community	General population	5/30/2020		X	X	X														X					X	
33611168	Cohabitation COVID-19 transmission rates in a United States suburban community: A retrospective study of familial infections	1/16/2021	D'Onofrio	C	Public Health	Analytic	Retrospective	Passive Surveillance	Community	General population	6/30/2020		X	X						X															
33622481	Severe Acute Respiratory Syndrome Coronavirus 2 Seropositivity among Healthcare Personnel in Hospitals and Nursing Homes, Rhode Island, USA, July-August 2020.	2/17/2021	Akinbami	B	EID	Analytic	Cross-sectional	Serosurvey	Skilled nursing facility	Healthcare Workers or First Responders	8/31/2020		X	X	X			X		X									X					X	
33630817	Suspected Recurrent SARS-CoV-2 Infections Among Residents of a Skilled Nursing Facility During a Second COVID-19 Outbreak - Kentucky, July-November 2020.	2/26/2021	Cavanaugh	B	MMWR	Descriptive	Case series or cluster	Field Data	Skilled nursing facility	LCTF residents and staff	12/7/2020																								
33630823	Clusters of SARS-CoV-2 Infection Among Elementary School Educators and Students in One School District - Georgia, December 2020-January 2021.	2/26/2021	Vallabhaneni	A	MMWR	Descriptive	Case series or cluster	Field Data	College or university	General population	1/22/2021																								
33630825	First Identified Cases of SARS-CoV-2 Variant B.1.1.7 in Minnesota - December 2020-January 2021	2/17/2021	Firestone	B	MMWR	Descriptive	Case series or cluster	Passive Surveillance	Community	General population	1/12/2021																								
33639620	Changes in Severe Acute Respiratory Syndrome Coronavirus 2 Seroprevalence Over Time in 10 Sites in the United States, March-August, 2020	2/26/2021	Lim	A	Clin Infect Dis	Analytic	Ecologic	Serosurvey	Community	General population	8/30/2020																X		X					X	
33661859	COVID-19 Outbreak Among Attendees of an Exercise Facility — Chicago, Illinois, August–September 2020	3/5/2021	Lendacki	B	MMWR	Analytic	Cross-sectional	Field Data	Gym or fitness facility	General population	9/15/2020		X				X																		
33661861	Community Transmission of SARS-CoV-2 at Three Fitness Facilities — Hawaii, June–July 2020	3/5/2021	Groves	B	MMWR	Descriptive	Case series or cluster	Field Data	Gym or fitness facility	General population	7/11/2020																								
33661862	Estimated SARS-CoV-2 Seroprevalence Among Persons Aged <18 Years — Mississippi, May–September 2020	3/5/2021	Hobbs	B	MMWR	Analytic	Cross-sectional	Serosurvey	Community	Children and Adolescents	9/19/2020		X	X														X	X					X	
33661864	SARS-CoV-2 Infection Risk Among Active Duty Military Members Deployed to a Field Hospital — New York City, April 2020	3/5/2021	Clifton	A	MMWR	Descriptive	Case series or cluster	Field Data	Healthcare facility	Armed Forces	4/30/2020																								
33688033	COVID-19 Infections Among Students and Staff in New York City Public Schools	3/9/2021	Varma	B	Pediatrics	Descriptive	Incidence	Passive Surveillance	Community	General population	12/18/2020																		X		X				
33705364	Association of State-Issued Mask Mandates and Allowing On-Premises Restaurant Dining with County-Level COVID-19 Case and Death Growth Rates — United States, March 1–December 31, 2020	3/12/2021	Guy	A	MMWR	Analytic	Ecologic	Passive Surveillance	Community	General population	12/31/2020		X				X																		
33705367	First Identified Cases of SARS-CoV-2 Variant P.1 in the United States — Minnesota, January 2021	4/3/2021	Firestone	B	MMWR	Descriptive	Case series or cluster	Passive Surveillance	Community	General population	1/25/2021																								
33705371	Body Mass Index and Risk for COVID-19–Related Hospitalization, Intensive Care Unit Admission, Invasive Mechanical Ventilation, and Death — United States, March–December 2020	3/12/2021	Kopmpaniyets	A	MMWR	Analytic	Cross-sectional	Medical records	Community	General population	12/30/2020											X			X										
33734836	Shelter Characteristics, Infection Prevention Practices, and Universal Testing for SARS-CoV-2 at Homeless Shelters in 7 US Urban Areas	3/18/2021	Self	B	AJPH	Analytic	Ecologic	Field Data	Homeless Facility	Homeless Individuals or Facility Staff	6/1/2020		X							X															
33735161	Minimal SARS-CoV-2 Transmission After Implementation of a Comprehensive Mitigation Strategy at a School — New Jersey, August 20–November 27, 2020	3/19/2021	Volpe	A	MMWR	Descriptive	Cross-sectional	Passive Surveillance	Primary or secondary school	Children and Adolescents	11/27/2020																		X						
33735163	Notes from the Field: SARS-CoV-2 Transmission Associated with High School Football Team Members — Florida, September–October 2020	3/19/2021	Siegel	C	MMWR	Descriptive	Case series or cluster	Field Data	Primary or secondary school	Children and Adolescents	10/30/2020																		X						
33735165	Racial and Ethnic Disparities in COVID-19 Incidence by Age, Sex, and Period Among Persons Aged <25 Years — 16 U.S. Jurisdictions, January 1–December 31, 2020	3/19/2021	Van Dyke	B	MMWR	Descriptive	Incidence	Passive Surveillance	Community	General population	12/31/2020																		X		X				
33754981	Characteristics and Risk Factors of Hospitalized and Nonhospitalized COVID-19 Patients, Atlanta, Georgia, USA, March–April 2020.	2/26/2021	Pettrone	B	EID	Analytic	Cross-sectional	Medical records	Community	General population	4/7/2020											X	X		X										
33762539	Excess Mortality Associated With the COVID-19 Pandemic-Los Angeles County, March-September 2020	5/1/2021	Traub	C	J Public Health Manag Pract	Descriptive	Incidence	Vital statistics	Community	General population	8/15/2020																		X						X
33764961	Pilot Investigation of SARS-CoV-2 Secondary Transmission in Kindergarten Through Grade 12 Schools Implementing Mitigation Strategies - St. Louis County and City of Springfield, Missouri, December 2020.	3/26/2021	Dawson	B	MMWR	Descriptive	Case series or cluster	Field Data	Primary or secondary school	General population	12/31/2020																								
33764962	COVID-19 in Primary and Secondary School Settings During the First Semester of School Reopening - Florida, August-December 2020.	3/19/2021	Doyle	B	MMWR	Analytic	Ecologic	Passive Surveillance	Community	Children and Adolescents	12/21/2020		X				X				X														
33764967	Low SARS-CoV-2 Transmission in Elementary Schools — Salt Lake County, Utah, December 3, 2020–January 31, 2021	3/26/2021	Hershow	B	MMWR	Descriptive	Case series or cluster	Field Data	Primary or secondary school	Children and Adolescents	1/31/2021																								
33793462	Rapid Spread of SARS-CoV-2 in a State Prison After Introduction by Newly Transferred Incarcerated Persons - Wisconsin, August 14-October 22, 2020.	4/2/2021	Hershow	B	MMWR	Descriptive	Case series or cluster	Field Data	Correctional or detention facility	Prison inmates and staff	10/20/2020																								
33793463	Counties with High COVID-19 Incidence and Relatively Large Racial and Ethnic Minority Populations — United States, April 1–December 22, 2020	4/2/2021	Lee	A	MMWR	Descriptive	Incidence	Passive Surveillance	Community	General population	12/22/2020																		X		X				
33793464	Community-Associated Outbreak of COVID-19 in a Correctional Facility — Utah, September 2020–January 2021	4/2/2021	Lewis	B	MMWR	Descriptive	Case series or cluster	Field Data	Correctional or detention facility	Prison inmates and staff	1/31/2021	X																							
33813042	Outbreak of COVID-19 and interventions in a large jail - Cook County, IL, United States, 2020	4/2/2021	Zawitz	B	Am J Infect Control	Descriptive	Case series or cluster	Field Data	Correctional or detention facility	Prison inmates and staff	4/30/2020																								
33830981	Community Transmission of SARS-CoV-2 Associated with a Local Bar Opening Event — Illinois, February 2021	4/9/2021	Sami	B	MMWR	Descriptive	Case series or cluster	Field Data	Community	General population	12/28/2021																								
33830986	COVID-19 Incidence and Mortality Among American Indian/Alaska Native and White Persons — Montana, March 13–November 30, 2020	4/9/2021	Williamson	C	MMWR	Descriptive	Incidence	Passive Surveillance	Community	General population	11/30/2020																		X		X			X	
33830988	Provisional Mortality Data — United States, 2020	4/9/2021	Ahmad	A	MMWR	Descriptive	Incidence	Passive Surveillance	Community	General population	12/31/2020																		X					X	
33835179	Characteristics and Disease Severity of US Children and Adolescents Diagnosed With COVID-19	4/9/2021	Preston	A	JAMA	Analytic	Retrospective	Administrative data	Community	Children and Adolescents	12/9/2020											X	X	X	X										

S3 Table: Abstracted Data From Reports Meeting Scoping Review Inclusion Criteria

33836067	Seroprevalence of Severe Acute Respiratory Syndrome Coronavirus 2 Following the Largest Initial Epidemic Wave in the United States: Findings From New York City, 13 May to 21 July 2020	4/9/2021	Pathela	A	J Infect Dis	Analytic	Cross-sectional	Serosurvey	Community	General population	7/21/2020				X	X	X	X		X		X		X																	X						X	
33856883	SARS-CoV-2 Infection Among Correctional Staff in the Federal Bureau of Prisons	4/15/2020	Toblin	A	AJPH	Analytic	Case series or cluster	Passive Surveillance	Correctional or detention facility	Prison inmates and staff	6/17/2020				X					X																												
33857062	Emergency Department Visits for COVID-19 by Race and Ethnicity - 13 States, October-December 2020.	4/16/2021	Smith	A	MMWR	Analytic	Ecologic	Active surveillance	Community	General population	12/31/2020				X	X	X																								X			X				
33857065	Notes from the Field: Update on Excess Deaths Associated with the COVID-19 Pandemic - United States, January 26, 2020-February 27, 2021.	4/16/2021	Rossen	A	MMWR	Analytic	Ecologic	Vital statistics	Community	General population	2/27/2021																																					X
33857067	COVID-19 Stats: COVID-19* and Influenza† Discharge Diagnoses as a Percentage of Emergency Department (ED) Visits,§ by Year - United States, June 2018-March 2021.	4/16/2021	None listed	A	MMWR	Descriptive	Incidence	Active surveillance	Community	General population	3/31/2021																														X			X				
33882112	Investigation and public health response to a COVID-19 outbreak in a rural resort community-Blaine County, Idaho, 2020	4/21/2021	Dunne	B	Plos One	Descriptive	Case series or cluster	Passive Surveillance	Community	General population	4/10/2020																																					
33886536	COVID-19 Outbreaks in Correctional Facilities with Work-Release Programs — Idaho, July–November 2020	4/23/2021	Dunne	B	MMWR	Descriptive	Case series or cluster	Passive Surveillance	Correctional or detention facility	Prison inmates and staff	11/30/2020																														X		X					
33900168	Epidemiologic Findings from Case Investigations and Contact Tracing for First 200 Cases of Coronavirus Disease, Santa Clara County, California, USA.	4/1/2021	Ortiz	B	EID	Descriptive	Case series or cluster	Passive Surveillance	Community	General population	3/20/2020																																					
33900171	SARS-CoV-2 in Nursing Homes after 3 Months of Serial, Facilitywide Point Prevalence Testing, Connecticut, USA.	4/1/2021	Ehrlich	C	EID	Analytic	Ecologic	Passive Surveillance	Skilled nursing facility	LCTF residents and staff	6/25/2020				X					X																												
33900189	SARS-CoV-2 Serial Interval Variation, Montana, USA, March 1–July 31, 2020.	4/15/2021	Reed	C	EID	Analytic	Retrospective	Passive Surveillance	Community	General population	7/31/2020		X																																			
33900192	Racial and Ethnic Disparities in Incidence of SARS-CoV-2 Infection, 22 US States and DC, January 1–October 1, 2020.	4/15/2021	Hollis	A	EID	Descriptive	Incidence	Passive Surveillance	Community	General population	10/1/2020																														X		X					
33904584	COVID-19 Hospitalizations Among U.S. Medicare Beneficiaries With Inflammatory Bowel Disease, April 1 to July 31, 2020	4/27/2021	Xu	A	Inflamm Bowel Dis	Analytic	Retrospective	Administrative data	Community	General population	7/31/2020														X			X																				
33912901	Coronavirus Disease 2019 (COVID-19) Outbreaks at 2 Construction Sites-New York City, October-November 2020	4/29/2021	Bushman	B	Clin Infect Dis	Descriptive	Case series or cluster	Field Data	Community	Other Occupations	10/29/2020																																					
33912907	Widespread Severe Acute Respiratory Syndrome Coronavirus 2 Transmission Among Attendees at a Large Motorcycle Rally and their Contacts, 30 US Jurisdictions, August-September, 2020	4/29/2021	Carter	B	Clin Infect Dis	Descriptive	Case series or cluster	Passive Surveillance	Social gathering	General population	9/30/2020																																					
33914719	COVID-19 Outbreak Among Farmworkers — Okanogan County, Washington, May–August 2020	4/30/21	Miller	B	MMWR	Analytic	Cross-sectional	Passive Surveillance	Other Workplace	Other Occupations	8/31/2020				X					X																												
33914720	COVID-19 Outbreak Associated with a SARS-CoV-2 R.1 Lineage Variant in a Skilled Nursing Facility After Vaccination Program — Kentucky, March 2021	4/21/2021	Cavanaugh	B	MMWR	Descriptive	Case series or cluster	Field Data	Skilled nursing facility	LCTF residents and staff	3/31/2021																																					
33914724	Linked Clusters of SARS-CoV-2 Variant B.1.351 — Maryland, January–February 2021	4/30/2021	Feder	B	MMWR	Descriptive	Case series or cluster	Field Data	Community	General population	2/28/2021																																					
33979564	Industry Sectors Highly Affected by Worksite Outbreaks of Coronavirus Disease, Los Angeles County, California, USA, March 19–September 30, 2020	7/1/2021	Contreras	B	EID	Descriptive	Case series or cluster	Field Data	Other Workplace	General population	9/30/2020																																					
33983915	Rapid Emergence and Epidemiologic Characteristics of the SARS-CoV-2 B.1.526 Variant — New York City, New York, January 1–April 5, 2021	5/14/2021	Thompson	C	MMWR	Analytic	Retrospective	Passive Surveillance	Community	General population	4/5/2021														X							X																
33988184	Identification of and Surveillance for the SARS-CoV-2 Variants B.1.427 and B.1.429 — Colorado, January–March 2021	5/5/2021	Webb	C	MMWR	Descriptive	Case series or cluster	Passive Surveillance	Community	General population	3/31/2021																																					
33993309	Morbidity and Mortality Among Adults Experiencing Homelessness Hospitalized With COVID-19	5/16/2021	Cha	A	J Infect Dis	Descriptive	Case series or cluster	Active surveillance	Community	Homeless Individuals or Facility Staff	5/31/2020																																					
34014908	Characteristics of COVID-19 Cases and Outbreaks at Child Care Facilities — District of Columbia, July–December 2020	5/21/2021	Jkim	B	MMWR	Descriptive	Incidence	Passive Surveillance	Other Workplace	Children and Adolescents	12/31/2020																														X		X					
34043610	Mask Use and Ventilation Improvements to Reduce COVID-19 Incidence in Elementary Schools — Georgia, November 16–December 11, 2020	5/28/2021	Gettings	B	MMWR	Analytic	Ecologic	Passive Surveillance	Primary or secondary school	Children and Adolescents	12/11/2020				X				X		X																											
34043706	Seroprevalence of SARS-CoV-2 antibodies in Seattle, Washington: October 2019-April 2020	5/27/2021	McCulloch	A	Plos One	Analytic	Cross-sectional	Serosurvey	Community	General population	4/1/2020																														X						X	
34081608	Census Tract Patterns and Contextual Social Determinants of Health Associated With COVID-19 in a Hispanic Population From South Texas: A Spatiotemporal Perspective	5/8/2021	Bauer	C	JMIR Public Health Surveill	Analytic	Retrospective	Passive Surveillance	Community	General population	12/16/2020				X									X																	X	X						
34086923	Prevalence of SARS-CoV-2 Antibodies in New York City Adults, June-October 2020: A Population-Based Survey.	6/4/2021	Parrott	B	JID	Analytic	Cross-sectional	Serosurvey	Community	General population	10/31/2020				X	X	X			X				X																	X						X	
34097050	Underlying Medical Conditions Associated With Severe COVID-19 Illness Among Children	6/7/2021	Kompaniyets	A	JAMA	Analytic	Retrospective	Administrative data	Community	Children and Adolescents	1/31/2021														X			X																				
34097664	Estimated SARS-CoV-2 Seroprevalence in Healthy Children and Those with Chronic Illnesses in the Washington Metropolitan Area as of October 2020	7/1/2021	Bahar	A	Pediatr Infect Dis J	Analytic	Cross-sectional	Serosurvey	Community	Children and Adolescents	10/29/2020				X	X	X	X				X		X																	X						X	
34111061	Hospitalization of Adolescents Aged 12–17 Years with Laboratory-Confirmed COVID-19 — COVID-NET, 14 States, March 1, 2020–April 24, 2021	6/11/2021	Havers	A	MMWR	Descriptive	Incidence	Passive Surveillance	Community	Children and Adolescents	4/24/2020																														X				X			
34111937	Excess Mortality During the COVID-19 Pandemic in Philadelphia	7/10/2021	Todd	C	AJPH	Descriptive	Incidence	Vital statistics	Community	General population	1/2/2021																																					X
34138695	Transmission Dynamics of Severe Acute Respiratory Syndrome Coronavirus 2 in High-Density Settings, Minnesota, USA, March–June 2020.	6/15/2021	Lehnertz	B	EID	Descriptive	Case series or cluster	Passive Surveillance	Congregate living facilities (multiple)	General population	6/30/2020																																					
34166336	COVID-19 Surveillance and Investigations in Workplaces — Seattle & King County, Washington, June 15–November 15, 2020	6/25/2021	Bonwit	B	MMWR	Analytic	Cross-sectional	Passive Surveillance	Other Workplace	Other Occupations	11/15/2020				X					X																												
34193337	Patterns of Virus Exposure and Presumed Household Transmission among Persons with Coronavirus Disease, United States, January–April 2020.	6/30/2021	Burke	B	EID	Analytic	Cross-sectional	Survey	Community	General population	4/4/2020	X			X							X																										
34197282	Risk for Acquiring Coronavirus Disease Illness among Emergency Medical Service Personnel Exposed to Aerosol-Generating Procedures.	7/1/2021	Brown	C	EID	Analytic	Retrospective	Administrative data	Community	Healthcare Workers or First Responders	7/31/2020				X					X																												
34264910	SARS-CoV-2 B.1.617.2 (Delta) Variant COVID-19 Outbreak Associated with a Gymnastics Facility — Oklahoma, April–May 2021	7/16/2021	Dougherty	C	MMWR	Descriptive	Case series or cluster	Field Data	Gym or fitness facility	Children and Adolescents	5/18/2021																																					
34346103	COVID-19 mortality among Amalgamated Transit Union (ATU) and Transport Workers Union (TWU) workers—March–July 2020, New York City metro area	8/3/2021	Tomasi	A	Am J Ind Med	Descriptive	Case series or cluster	Field Data	Community	Other Occupations	7/31/2020																																					
34383734	Rapid Increase in Circulation of the SARS-CoV-2 B.1.617.2 (Delta) Variant — Mesa County, Colorado, April–June 2021	8/16/2021	Herlihy	B	MMWR	Descriptive	Cross-sectional	Passive Surveillance	Community	General population	6/26/2021																														X							
34399085	Severe Acute Respiratory Syndrome Coronavirus 2 Transmission in Georgia, USA, February 1–July 13, 2020.	8/16/2021	Wang	A	EID	Analytic	Retrospective	Passive Surveillance	Community	General population	7/13/2020		X	X																																		
34411075	Disparities in Excess Mortality Associated with COVID-19 - United States, 2020.	8/20/2021	Rossen	A	MMWR	Analytic	Ecologic	Vital statistics	Community	General population	1/2/2021																																					X
34433659	SARS-CoV-2 antibody seroprevalence among firefighters in Orange County, California.	8/25/2021	Vieira	C	Occup Environ Med	Analytic	Cross-sectional	Serosurvey	Community	Healthcare Workers or First Responders	10/31/2020				X					X																					X						X	
34463672	Outbreak Among a University's Men's and Women's Soccer Teams — Chicago, Illinois, July–August 2020.	10/30/2020	Teran	B	MMWR	Analytic	Case control	Field Data	College or university	College students	8/30/2020				X				X			X																										
34473679	COVID-19 Case Rates in Transitional Kindergarten Through Grade 12 Schools and in the Community — Los Angeles County, California, September 2020–March 2021	9/3/2021	Yin	C	MMWR	Descriptive	Ecologic	Passive Surveillance	Primary or secondary school	Children and Adolescents	3/31/2020																														X							
34473681	Epidemiologically Linked COVID-19 Outbreaks at a Youth Camp and Men's Conference — Illinois, June–July 2021	8/31/2021	Matthias	B	MMWR	Descriptive	Case series or cluster	Field Data	Social gathering	General population	7/31/2021																																					
34473683	Outbreak Associated with SARS-CoV-2 B.1.617.2 (Delta) Variant in an Elementary School — Marin County, California, May–June 2021	9/3/21	Lam-Hine	C	MMWR	Descriptive	Case series or cluster	Field Data	Primary or secondary school	Children and Adolescents	5/31/2021																																					
34473687	Multiple Variants of SARS-CoV-2 in a University Outbreak After Spring Break - Chicago, Illinois, March-May 2021.	9/3/2021	Doyle	B	MMWR	Descriptive	Case series or cluster	Field Data	College or university	College students	4/5/2021																																					
34473791	Risk factors for hospitalization among persons with COVID-19-Colorado	9/2/2021	Vahey	B	Plos One	Analytic	Retrospective	Passive Surveillance	Community	General population	3/31/2020														X	X	X	X	X																			
34499627	Hospitalizations Associated with COVID-19 Among Children and Adolescents — COVID-NET, 14 States, March 1, 2020–August 14, 2021	9/10/2021	Delahoy	B	MMWR	Descriptive	Retrospective	Passive Surveillance	Community	Children and Adolescents	8/14/2021																														X							
34499628	[illegible] Department Visits, and Hospital Admissions Among Children and Adolescents Aged 0–17 Years — United States, August 2020–August	9/10/2021	Siegel	A	MMWR	Descriptive	Incidence	Passive Surveillance	Community	Children and Adolescents	8/27/2021																														X		X		X			
34529634	Disaggregating Data to Measure Racial Disparities in COVID-19 Outcomes and Guide Community Response - Hawaii, March 1, 2020-February 28, 2021.	9/17/2021	Quint	B	MMWR	Descriptive	Case series or cluster	Passive Surveillance	Community	General population	2/28/2021																														X		X			X		
34529634	Disaggregating Data to Measure Racial Disparities in COVID-19 Outcomes and Guide Community Response — Hawaii, March 1, 2020–February 28, 2021	9/17/2021	Quint	C	MMWR	Descriptive	Incidence	Passive Surveillance	Community	General population	2/26/2021																														X		X		X	X		
34545792	Predictors of Test Positivity, Mortality, and Seropositivity during the Early Coronavirus Disease Epidemic, Orange County, California, USA.	8/18/2021	Parker	C	EID	Analytic	Retrospective	Serosurvey	Community	General population	8/16/2020				X	X	X							X	X	X	X						X															
34545794	Multiple Transmission Chains within COVID-19 Cluster, Connecticut, USA, 2020.	8/23/2021	Bart	B	EID	Descriptive	Case series or cluster	Field Data	Community	General population	10/30/2020																																					
34545796	Characteristics, Comorbidities, and Data Gaps for Coronavirus Disease Deaths, Tennessee, USA.	8/23/2021	Parker	C	EID	Analytic	Retrospective	Medical records	Community	General population	8/15/2020														X	X	X	X																				
34586058	Interventions to Disrupt Coronavirus Disease Transmission at a University, Wisconsin, USA, August–October 2020.	9/24/2021	Currie	B	EID	Descriptive	Case series or cluster	Passive Surveillance	College or university	College students	10/31/2020																																					
34591829	Pediatric COVID-19 Cases in Counties With and Without School Mask Requirements — United States, July 1–September 4, 2021	10/21/2021	Budzyn	A	MMWR	Analytic	Ecologic	Passive Surveillance	Community	Children and Adolescents	9/4/2021				X				X																													
34591830	Association Between K–12 School Mask Policies and School-Associated COVID-19 Outbreaks — Maricopa and Pima Counties, Arizona, July–August 2021	10/1/2021	Jehn	B	MMWR	Analytic	Ecologic	Passive Surveillance	Primary or secondary school	Children and Adolescents	8/31/2021				X				X																													
34612108	Is Symptom Screening Useful for Identifying COVID-19 Infection in School Settings? Georgia, USA.	10/4/2021	Swanson	B	J Sch Nursing	Analytic	Cross-sectional	Field Data	Primary or secondary school	Children and Adolescents	1/22/2021																							X														

S3 Table: Abstracted Data From Reports Meeting Scoping Review Inclusion Criteria

34618799	COVID-19 Outbreaks at Youth Summer Camps — Louisiana, June–July 2021	10/8/2021	Tonzel	C	MMWR	Descriptive	Incidence	Passive Surveillance	Social gathering	Children and Adolescents	7/25/2021																													x		x				
34618801	Distribution of SARS-CoV-2 Variants in a Large Integrated Health Care System - California, March-July 2021	10/8/2021	Malden	A	MMWR	Analytic	Retrospective	Medical records	Community	General population	7/21/2021														X							x															
34637377	Mask effectiveness for preventing secondary cases of COVID-19, Johnson County, Iowa, USA.	10/12/2021	Riley	C	EID	Analytic	Prospective	Field Data	Community	General population	3/1/2021	X			X				X																												
34649001	Protective Immunity after Natural Infection with Severe Acute Respiratory Syndrome Coronavirus-2 (SARS-CoV-2) - Kentucky, USA, 2020	10/12/2021	Spicer	B	Int J Infect Dis	Analytic	Retrospective	Passive Surveillance	Community	General population	12/31/2020				X	X							X																								
34670651	Association of Shared Living Spaces and COVID-19 in University Students, Wisconsin, USA, 2020	9/22/2021	Bigouette	B	EID	Analytic	Retrospective	Administrative data	College or university	College students	12/19/2020				X							X																									
34673962	Racial and Ethnic Disparities in Rates of COVID-19-Associated Hospitalization, Intensive Care Unit Admission, and In-Hospital Death in the United States From March 2020 to February 2021	10/21/2021	Acosta	B	JAMA	Analytic	Cross-sectional	Active surveillance	Community	General population	2/28/2021														X	X	X												x		x				x		
34710076	Severity of Disease Among Adults Hospitalized with Laboratory-Confirmed COVID-19 Before and During the Period of SARS-CoV-2 B.1.617.2 (Delta) Predominance — COVID-NET, 14 States, January–August 2021	10/29/2021	Taylor	B	MMWR	Descriptive	Incidence	Passive Surveillance	Community	General population	8/31/2021																													x				x	x	
34736612	SARS-CoV-2 Testing Before International Airline Travel, December 2020 to May 2021	9/1/2021	Tande	C	Proc Mayo Clin	Descriptive	Case series or cluster	Field Data	Community	General population	5/19/2021																																				
34752156	Assessment of SARS-CoV-2 Seroprevalence by Community Survey and Residual Specimens, Denver, Colorado, July-August 2020	11/9/2021	Kugeler	B	Public Health Rep	Analytic	Cross-sectional	Serosurvey	Community	General population	7/30/2020				X	X	X			X		X																		x						x
34761531	Community predictors of COVID-19 cases and deaths in Massachusetts: Evaluating changes over time using geospatially refined data	11/10/2021	Spangler	C	Influenza Other Respir Viruses	Analytic	Ecologic	Passive Surveillance	Community	General population	2/28/2021				X									X																							
34770012	Assessment of Retrospective COVID-19 Spatial Clusters with Respect to Demographic Factors: Case Study of Kansas City, Missouri, United States	11/1/2021	AlQuadi	C	Int J Environ Res Public Health	Analytic	Ecologic	Passive Surveillance	Community	General population	11/30/2020				X									X																							
34793690	SARS-CoV-2 infections, including COVID-19 vaccine breakthrough infections, associated with large public gatherings, United States.	11/18/2021	Gharpure	B	EID	Descriptive	Case series or cluster	Field Data	Community	General population	8/10/2021																																				
34924621	SARS-CoV-2 Reinfections during the Delta Variant Surge - Missouri, June-October, 2021	11/30/2021	Turabelidze	C	Mo Med	Descriptive	Incidence	Passive Surveillance	Community	General population	10/17/2021																													x		x				
32820074	Characterizing superspreading events and age-specific infectiousness of SARS-CoV-2 transmission in Georgia, USA	8/20/2020	Lau	C	Proc Natl Acad Sci USA	Analytic	Retrospective	Passive Surveillance	Community	General population	5/3/2020			X																																	
