# Supplementary material for: Missing science: A scoping study of COVID-19 epidemiological data in the United States
Source: PLoS One. 2022 Oct 12;17(10):e0248793. doi: 10.1371/journal.pone.0248793 (PMC9555641; doi:10.1371/journal.pone.0248793)
Supplement: Attachment — Submitted filename: PONE-D-21-07710_Reviewer_Comments.pdf [file pone.0248793.s004.pdf]

This is a much needed and important article. The public health data collection efforts in the United States have been incredibly fragmented. This, though, leads me to my main critiques of the paper.

Overall
The authors state in the conclusion that:
"CDC scientists have the access to data, the expertise, and the resources to provide the data necessary for an optimal epidemic response."

This is actually not entirely true. Public health data collection in the United States is heavily fragmented. In fact, many states did not report or provide the CDC with their COVID-19 surveillance data. This was further exacerbated by the lack of data sharing between health systems and hospitals, as well as, between hospitals and departments of public health. I think that adding a more nuanced, in-depth discussion of why the CDC might not have updated domestic data (or why we still have COVID-19 data problems over a year later) could make this article stronger. Despite the July 2020 HHS requirement, data collection was in fact a bit of a mess and extremely cumbersome. Refer to (pg. 13 provides a few examples): https://oig.hhs.gov/oei/reports/OEI-09-21-00140.pdf

Additionally see: Galaitsi, S.E., Cegan, J.C., Volk, K., Joyner, M., Trump, B.D. and Linkov, I., 2021. The challenges of data usage for the United States' COVID-19 response. *International Journal of Information Management*, *59*, p.102352.

Perhaps, the CDC should place more thought into how data is collected and who is in charge of establishing the back end for data collection. Alternatively, how do you deal with situations where state and federal requirements do not align but instead cause increased administrative burdens?

If fact, it might explain a very well received point the authors make: "The content of their [the CDC's] investigations raises questions about whether and how an explicit national research agenda guided CDC epidemiological endeavors…" as well as, "Regional differences in prevalence could explain part of the variation in the pace of infectious spread." Politics and federalism clearly play/played a role in data collection and reporting (Florida and Nebraska anyone?), as has been discussed in multiple published articles this last year. For example, see:

Rocco, P., Rich, J.A., Klasa, K., Dubin, K.A. and Béland, D., 2021. Who Counts Where? COVID-19 Surveillance in Federal Countries. *Journal of Health Politics, Policy and Law*.

Additionally, the authors could suggest (and cite existing literature) about how to best go about data collection for the specific parameters that they identify. For example, are there essential occupations that we know have had more exposure risk or that peer-reviewed articles have shown to have higher transmission rates? Is there perhaps a

region, state, or city that was a stellar example of data collection and data-informed policy decisions? What did they do correct?

I think that more thought should be placed on *who* is collecting the data (or should be in charge of collecting the data). The authors infer that this is the CDC, but what challenges might the CDC face in collecting data? And if it is inevitably going to face difficulties then how can we make data collection most effective (and ensure health equity)? What does the evidence say that worked this last year and what didn't work? Right now, this reads like a giant list of wants in a perfect world. But, these data might not all be possible (or feasible or cost effective) to collect.

Discussion (general)
I think that the authors could cite existing studies in peer-reviewed literature that do exist whenever they mention that "x" doesn't exist or "y" doesn't exist. I realize that the authors limited their search to only studies with CDC affiliated authors, but I think that it is important to emphasize that local (city, county) and state efforts did exist that tried to figure this data out because the CDC was very slow to provide good data throughout 2020. There were studies conducted on infectious transmission, disease severity, burden of infection/disease in smaller settings since COVID-19 began (just not with CDC affiliated investigators). It might be important to cite a few of these throughout the discussion and note that the CDC might have determined that the few US studies that do exist were too small for them to consider. There were some universities that partnered with state or local departments of public health that did some good work.

Infectious Transmission Section
- Could you cite a few studies that do exist that tried to quantify infectious transmission? It would be useful to reference what the 'best available" study that exists is (for example, 'no identified transmission studies have quantitatively examined….then what are the qualitative studies that exist?)
- Are the restaurant restrictions not because of the believe that the virus is airborne?
- Would state-to-state variability in policy matter at the community level as much as county-to-county variation?
- Many employers do not report COVID-19 infections.
- Contact tracing will require community trust – even in places with robust contact tracing efforts, follow-up was often difficult or challenging (especially in low-income or immigrant communities)
- Some household transmission studies were conducted. For example, Lewis, N.M., Chu, V.T., Ye, D., Conners, E.E., Gharpure, R., Laws, R.L., Reses, H.E., Freeman, B.D., Fajans, M., Rabold, E.M. and Dawson, P., 2020. Household transmission of SARS-CoV-2 in the United States. *Clinical infectious diseases: an official publication of the Infectious Diseases Society of America*.

Disease Severity Section

- In the second sentence, did you mean “county to country” differences or “county to county” differences or “country to country” differences?

Minor Edits

Last, there are some minor edits that need to be made:
- There are a few grammatical errors throughout that shouldn’t be too difficult to fix after another read through
- Citations need to be fixed and standardized
- Make sure to add updated citations and engage with more recent literature
