# Supplementary material for: Missing science: A scoping study of COVID-19 epidemiological data in the United States
Source: PLoS One. 2022 Oct 12;17(10):e0248793. doi: 10.1371/journal.pone.0248793 (PMC9555641; doi:10.1371/journal.pone.0248793)
Supplement: Attachment — Submitted filename: PONE-D-21-07710_Reviewer_Comments_2.pdf [file pone.0248793.s006.pdf]

Thank you so much for letting me review this paper. I can tell that a huge amount of effort and work went into your revisions. Table 3 and Table 4 are excellent! Table 5 could be a table or a figure (if a figure is easier to make with just the list of questions in a box or something like that). The paper is excellent, informative, and a great contribution to public health research! I do not think that any more substantive edits need to be made. Fantastic job ☺

If there is time, then there are only a few minor grammar edits to be made throughout. A quick read through with fresh eyes should catch all of them.

For example:

Page 6, paragraph 2: "….Our aims were to assess the completeness of this data during the first two years of the pandemic-19…"

I believe you wanted to stay "COVID-19 pandemic" or "pandemic caused by the Sars-COV-2 virus".

Page 25, paragraph 3: "Furthermore, also did not examine or judge…"

*I believe you wanted to say "**we** also did not examine…"*

Page 26, paragraph 3: "While many epidemiologic questions COVID-19 required…."

*I believe you wanted to say "While many epidemiologic questions **about** COVID-19…"*
