# Supplementary material for: Missing science: A scoping study of COVID-19 epidemiological data in the United States
Source: PLoS One. 2022 Oct 12;17(10):e0248793. doi: 10.1371/journal.pone.0248793 (PMC9555641; doi:10.1371/journal.pone.0248793)
Supplement: Attachment — Submitted filename: covid-scoping-review-reviewer1-response.docx [file pone.0248793.s005.docx]

Authors’ Response to Reviewer 1

C1: *The authors state in the conclusion that: “CDC scientists have the access to data, the expertise, and the resources to provide the data necessary for an optimal epidemic response.”This is actually not entirely true. Public health data collection in the United States is heavily fragmented. In fact, many states did not report or provide the CDC with their COVID-19 surveillance data. This was further exacerbated by the lack of data sharing between health systems and hospitals, as well as, between hospitals and departments of public health. I think that adding a more nuanced, in-depth discussion of why the CDC might not have updated domestic data (or why we still have COVID-19 data problems over a year later) could make this article stronger. Despite the July 2020 HHS requirement, data collection was in fact a bit of a mess and extremely cumbersome. Refer to (pg. 13 provides a few examples):* [*https://oig.hhs.gov/oei/reports/OEI-09-21-*](https://oig.hhs.gov/oei/reports/OEI-09-21-) *00140.pdf
Additionally see: Galaitsi, S.E., Cegan, J.C., Volk, K., Joyner, M., Trump, B.D. and
Linkov, I., 2021. The challenges of data usage for the United States’ COVID-19
response. International Journal of Information Management, 59, p.102352.
Perhaps, the CDC should place more thought into how data is collected and who is in
charge of establishing the back end for data collection. Alternatively, how do you deal
with situations where state and federal requirements do not align but instead cause
increased administrative burdens?
If fact, it might explain a very well received point the authors make: “The content of their
[the CDC’s] investigations raises questions about whether and how an explicit national
research agenda guided CDC epidemiological endeavors…” as well as, “Regional
differences in prevalence could explain part of the variation in the pace of infectious
spread.” Politics and federalism clearly play/played a role in data collection and
reporting (Florida and Nebraska anyone?), as has been discussed in multiple published
articles this last year. For example, see:
Rocco, P., Rich, J.A., Klasa, K., Dubin, K.A. and Béland, D., 2021. Who Counts Where?
COVID-19 Surveillance in Federal Countries. Journal of Health Politics, Policy and Law.
Additionally, the authors could suggest (and cite existing literature) about how to best go
about data collection for the specific parameters that they identify. For example, are
there essential occupations that we know have had more exposure risk or that peer reviewed
articles have shown to have higher transmission rates? Is there perhaps a
region, state, or city that was a stellar example of data collection and data-informed
policy decisions? What did they do correct?
I think that more thought should be placed on who is collecting the data (or should be in
charge of collecting the data). The authors infer that this is the CDC, but what
challenges might the CDC face in collecting data? And if it is inevitably going to face
difficulties then how can we make data collection most effective (and ensure health
equity)? What does the evidence say that worked this last year and what didn’t work?
Right now, this reads like a giant list of wants in a perfect world. But, these data might
not all be possible (or feasible or cost effective) to collect.*

R1: We have added a new sub-section in the discussion to discuss possible explanations for the gaps we observed in public health agency surveillance data and original investigations dependent on that data which includes discussion of the lack of standardized surveillance data collection and reporting protocols. We note, however, that many epidemiological investigations utilized data sources that not derived from public disease surveillance systems. And many of the research gaps we identify might have been addressed with the application of well-established method (e.g. community cohort studies).

*C2: I think that the authors could cite existing studies in peer-reviewed literature that do
exist whenever they mention that “x” doesn’t exist or “y” doesn’t exist. I realize that the
authors limited their search to only studies with CDC affiliated authors, but I think that it
is important to emphasize that local (city, county) and state efforts did exist that tried to
figure this data out because the CDC was very slow to provide good data throughout
2020. There were studies conducted on infectious transmission, disease severity,
burden of infection/disease in smaller settings since COVID-19 began (just not with
CDC affiliated investigators). It might be important to cite a few of these throughout the
discussion and note that the CDC might have determined that the few US studies that
do exist were too small for them to consider. There were some universities that
partnered with state or local departments of public health that did some good work.*

R2: We concur. We expanded the scope of our review to include all published reports affiliated either with the US CDC or with any US state or local public health agencies. Most included studies were conducted and authored by collaborations of federal, state, and local governmental public health actors. We also added a new sub-section in the discussion that describes the contributions of U.S. public health affiliated publications to issue-specific COVID-19 epidemiological reviews.

*C3: Could you cite a few studies that do exist that tried to quantify infectious
transmission? It would be useful to reference what the ‘best available” study that
exists is (for example, ‘no identified transmission studies have quantitatively
examined….then what are the qualitative studies that exist?)*

R3. We included several U.S. public health affiliated studies that quantified the household secondary attack rate in the revision. We referenced a review of studies of the household secondary attack rate that demonstrates the international scope of research on the topic.

*C4: Are the restaurant restrictions not because of the believe that the virus is
airborne?*

R4: We removed the mention on restaurant restrictions specifically from the revised manuscript. We note that restrictions on in-person restaurant dining occurred before there existed either public health agency acknowledgement of airborne transmission.

*C5: Would state-to-state variability in policy matter at the community level as much
as county-to-county variation?*

R5: We agree that studying the variation local policy would be optimal.

*C6: Many employers do not report COVID-19 infections.*

R6: We agree. However, health care facilities and laboratory providers are subject to statutory requirements report cases under U.S. law. Employers do not have this statutory responsibility nor the ability, in most cases, the ability to assess COVID-19 infection status among their employees. However, case surveillance reports submitted by healthcare and utilized by public health agencies do include fields for occupation, industry, and work location. We acknowledge that morbidity report forms submitted to State and Federal public health agencies varied in completeness.

*C7: Contact tracing will require community trust – even in places with robust contact
tracing efforts, follow-up was often difficult or challenging (especially in low income
or immigrant communities)*

R7: We agree. In the revision, we have noted the challenges public health agencies faced in gaining participation from cases and contacts during disease investigations.

*C8: Some household transmission studies were conducted. For example, Lewis, N.M., Chu, V.T., Ye, D., Conners, E.E., Gharpure, R., Laws, R.L., Reses, H.E., Freeman, B.D., Fajans, M., Rabold, E.M. and Dawson, P., 2020. Household transmission of SARS-CoV-2 in the United States. Clinical infectious diseases: an official publication of the Infectious Diseases Society of America.*

R8: The above study by Lewis et al. was included in our initial review. We include several US additional household transmission studies in the updated review.

*C9: In the second sentence, did you mean “county to country” differences or “county
to county” differences or “country to country” differences?*

R9: We have revised the sentence to clarify.

*C10: There are a few grammatical errors throughout that shouldn’t be too difficult to fix
after another read through*

R10: Noted.

*C11: References are in non-standard formats.*

R11: All References are now standardized to NLM formats

*C12: Make sure to add updated citations and engage with more recent literature*

R12: The revision extends the review of authoritative estimates and published public health affiliated reports through Nov 30, 2021.
